# Molecularly self‐fueled nano-penetrator for nonpharmaceutical treatment of thrombosis and ischemic stroke

**DOI:** 10.1038/s41467-023-35895-5

**Published:** 2023-01-17

**Authors:** Hongyuan Zhang, Zhiqiang Zhao, Shengnan Sun, Sen Zhang, Yuequan Wang, Xuanbo Zhang, Jin Sun, Zhonggui He, Shenwu Zhang, Cong Luo

**Affiliations:** 1 Department of Pharmaceutics, Wuya College of Innovation, Shenyang Pharmaceutical University, Shenyang, 110016 P. R. China; 2 Key Laboratory of Structure-Based Drug Design and Discovery of Ministry of Education, Shenyang Pharmaceutical University, Shenyang, 110016 P. R. China

**Keywords:** Drug delivery, Thrombosis, Drug delivery

## Abstract

Thrombotic cerebro-cardiovascular diseases are the leading causes of disability and death worldwide. However, current drug therapeutics are compromised by narrow therapeutic windows, unsatisfactory thrombolysis effects, severe bleeding events, and high recurrence rates. In this study, we exploit a self-propelling nano-penetrator with high fuel loading and controllable motion features, which is molecularly co-assembled using a photothermal photosensitizer (DiR) and a photothermal-activable NO donor (BNN6). The precisely engineered nano-penetrator of the BNN6-DiR fuel pair shows distinct advantages in terms of NO productivity and autonomous motion under laser irradiation. In animal models of artery/vein thrombosis and acute ischemic stroke, the self‐fueled nano-penetrator enables self-navigated thrombus-homing accumulation, self-propelled clot deep penetration, fluorescence image-guided photothermal/mechanical thrombolysis, and NO-mediated prevention of thrombosis recurrence and acute ischemic stroke salvage. As expected, the molecularly self-fueled nano-penetrator displayed favorable therapeutic outcomes without bleeding risk compared to the clinically available thrombolytic drug. This study offers a facile, safe, and effective nonpharmaceutical modality towards the clinical treatment of thrombosis and ischemic stroke.

## Abstract (web summary)

Thrombotic cerebro-cardiovascular diseases are the leading causes of disability and death worldwide but current drug therapeutics show important limitations. Here, the authors exploit a selfpropelling nano-penetrator with high fuel loading and controllable motion which is molecularly co-assembled using a photothermal photosensitizer and a photothermal-activable NO donor.

## Introduction

Thrombotic cerebro-cardiovascular disorders have long been one of the most severe diseases with high morbidities and mortalities worldwide^1^. In particular, thrombotic ischemic stroke accounts for >80% of the total stroke events^2^. Thrombosis in blood vessels causes severe ischemic tissue injury and organ failures, leading to long-term disability and death^3,4^. Simultaneous recanalization of the blood vessels, restoration of the blood supply, and alleviation of the ischemic injury are of crucial importance in thrombotic disease interventions^5^. Currently, thrombolysis and neuroprotection remain the primary treatment options for embolism and thrombotic ischemic stroke, whereas clinical drug therapeutics are still far from satisfactory^6^. Notably, high doses of thrombolytic agents are commonly used for efficient thrombolysis owing to their short half-life and low utilization of most drugs, which inescapably disrupts the homeostasis balance of the blood-brain barrier with an increased risk of intracranial hemorrhage^7^. Moreover, there is still a lack of comprehensive therapeutic modalities for synchronous implementation of blood vessel recanalization in embolism lesions and blood flow reperfusion to ischemic areas.

Biomedical nanotechnology has provided unprecedented prospects for precise drug delivery^8^. The rational design of nanomedicines can effectively address the existing defects of antithrombotic drugs, such as improving the inferior physicochemical properties of drugs, extending the circulation time in the blood, reducing the off-target distribution in the body, and facilitating site-specific drug release in blood clots^9,10^. However, despite these significant advantages, the clinical translation of nanomedicines is still substantially impeded by inefficient drug loading, premature drug leakage, and carrier material-related biosafety risks^11^. Moreover, the encapsulation of thrombolytic drugs into conventional nanocarriers falls short of expectation as a solution for effective thrombolysis with low bleeding risk, due to off-target distribution and premature drug leakage prior to thrombus lesions^12^. Furthermore, most drugs or nanomedicines were located on the superficial surface of blood clots or ischemia areas with a short tissue penetration distance, leading to unsatisfactory therapeutic effects^13^. Significantly, the secondary recurrence of thrombus after treatment has become a severe challenge for thrombus therapy^14^. As a result, antithrombotic nanomedicines have not been approved for clinical application.

Nonpharmaceutical modalities such as photothermal therapy (PTT) and mechanotherapy have emerged as promising antithrombotic strategies in recent years^15^. It is worth noting that photothermal photosensitizers can be employed for image-guided hyperthermal thrombolysis, and effectively promote the deep penetration of drugs into the thrombi^9^. However, PTT alone is not sufficient for thrombus eradication and is accompanied by high recurrence rates of thrombosis^16^. In addition to PTT, micro/nanomotors (MNMs)-driven mechanotherapy has garnered considerable attention as an intelligent and controllable nonpharmaceutical modality, which has shown clear advantages in breaking through multiple biological barriers by mechanical motion in vivo^17^. Particularly, gas bubble-driven nanomotors greatly inspired research interests in biomedical applications by virtue of nano-size effects and good biocompatibility, such as nitric oxide (NO), oxygen (O_2_), and hydrogen (H_2_)^18^. Notably, some gases can serve as both driving forces and biological mediators in the body^19^. Given that NO contributes to thrombosis prevention and neuroprotection by improving endothelial function and relieving oxidative stress, NO-driven nanomotors are of great interest in the therapy of cerebro-cardiovascular diseases^20^. However, many shortcomings still impeded the clinical translation of conventional NO-driven nanomotors. On the one hand, most Janus nanomotors with asymmetric nanostructures tend to be readily phagocytized by the reticuloendothelial system (RES) and cleared from the body after intravenous injection^21,22^. However, most NO-driven nanomotors were fabricated by embedding NO donors in nanocarriers, resulting in inefficient fuel loading, NO production, and autonomous motion^23^. In addition, the motion efficiency of nanomotors consisting of endogenous stimuli-activatable NO donors can inevitably be affected by the heterogeneity of pathological microenvironments, such as l-arginine^24^, DETANONOate^25^, and S-nitrosothiols (RSNOs)^26^.

**Fig. 1:**
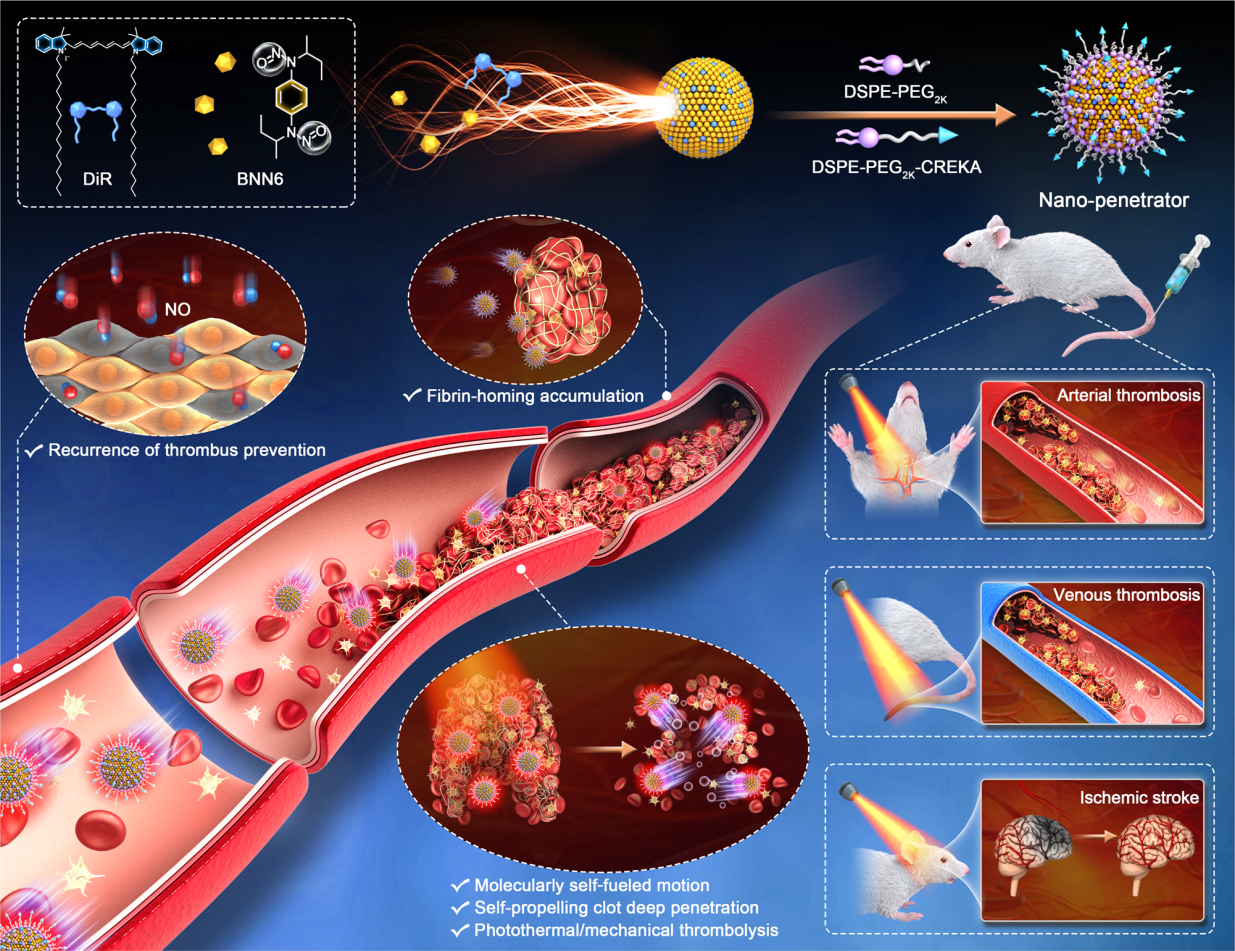
Schematic of molecularly self-fueled nano-penetrator for nonpharmaceutical treatment of thrombotic diseases. The molecularly engineered nano-penetrator was self-assembled by BNN6-DiR fuel pair without the assistance of any carrier materials. After PEGylation and fibrin-homing decoration, such a self-navigated, self‐fueled, and self-propelled nano-penetrator displayed satisfactory therapeutic outcomes in artery/vein thrombosis and acute ischemic stroke animal models.

This study proposed a precision nonpharmaceutical modality by elaborately engineering a carrier-free self-assembled nanomotor consisting of a photothermal photosensitizer and a photothermal-activable NO donor. As shown in Fig. 1, a novel nanoassembly was fabricated based on the co-assembly of 1,1’-dioctadecyl-3,3,3’,3’-tetramethylindotricarbocyanine iodide (DiR) and N, N’-di-sec-butyl-N, N’-dinitroso-1,4-phenylenediamine (BNN6). Notably, the BNN6/DiR fuel pair readily co-assembled into stable nanoassemblies with a wide range of BNN6/DiR molar ratios from 10:1 to 1:10. After engineering optimization, the preferred ratio of 1:3 (DiR/BNN6) was determined by comprehensively evaluating the nanoassembly features and NO generation efficiency. Under laser irradiation, the BNN6-DiR fuel pair acted as the power source to induce continuous changes in the momentum of the gas bubbles, which drove the vigorous motion of the fuel pair-engineered nanoassembly for deep clot penetration. We named this molecularly self-fueled nano-penetrator. After PEGylation and fibrin-homing decoration, the self-fueled nano-penetrator had impressively high fuel loading rates of DiR (41.1%) and BNN6 (33.9%). Therefore, 75% of the components served as fuel in the nano-penetrator, which was much higher than any previously reported nanomaterial-based nanomotors. With impressively high fuel loading capacity, the nano-penetrator displayed extraordinary NO generation and autonomous motion when compared to poly (lactic-co-glycolic acid) nanoparticles (PLGA NPs) loading with an equal proportion of DiR and BNN6. As expected, it demonstrated satisfactory therapeutic effect and favorable security in three animal models of thrombotic diseases when compared to lumbrokinase (LBK, a clinical first-line thrombolytic drug). In addition to thrombolysis, NO released from the nano-penetrator participates in thrombosis recurrence prevention and ischemic stroke relief by increasing cyclic guanosine monophosphate (GMP), downregulating platelets aggregation, remediating the microvasculature network around the lesions, and restoring blood flow to ischemic regions. To our knowledge, this is the first attempt to construct carrier-free photothermal nanomotor based on the interesting molecular nanoassembly of a fuel pair. Such a versatile nano-penetrator drives a conceptual step forward in gas-driven nanomotors.

## Results and discussion

### Elaborate engineering of a fuel pair nano-penetrator

**Fig. 2:**
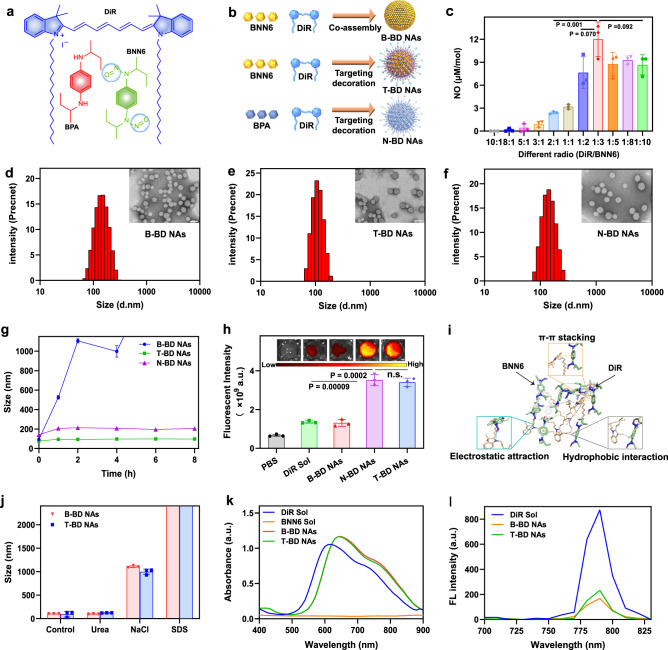
Elaborate engineering and characterization of fuel pair nano-penetrator. **a** Chemical structure of DiR, BNN6 (NO donor), and BPA (BNN6 precursor without NO production ability). **b** Co-assembly process of the binary NAs. **c** NO generation from B-BD NAs at various molar ratios of DiR and BNN6 from 10:1 to 1:10. Data are presented as mean ± SD (*n* = 3, independent experiments). Source data are provided as a Source Data file. One-way ANOVA (one-sided) with Dunnett’s multiple comparisons test was used for the analysis of data and adjusted *P* value. **d**–**f** Particle sizes distribution profiles and TEM images of B-BD NAs, T-BD NAs, and N-BD NAs, respectively, (Scale bar = 100 nm). Experiment was repeated three times independently with similar results. **g** Colloidal stability of B-BD NAs, T-BD NAs, and N-BD NAs incubated in PBS (pH 7.4) within 8 h at 37 ° C. Data are presented as mean ± SD (*n* = 3, independent experiments). **h** In vitro thrombus-targeting fluorescence intensity in artificial blood clots treated with PBS (pH 7.4), DiR Sol, B-BD NAs, N-BD NAs, and T-BD NAs, respectively. Data are presented as mean ± SD (*n* = 3, independent experiments). One-way ANOVA (one-sided) with Dunnett’s multiple comparisons test was used for the analysis of data and adjusted *P* value. The n.s. represent no significance. **i** Molecular docking simulation results of BNN6 and DiR. **j** Particle size changes of B-BD NAs and T-BD NAs in the presence of urea (50 mM), NaCl (50 mM), and SDS (50 mM), respectively (*n* = 3, independent experiments). **k** UV absorption spectra of DiR Sol, BNN6 Sol, B-BD NAs, and T-BD NAs. **l** Fluorescence spectra of DiR Sol, B-BD NAs, and T-BD NAs.

This project started with an interesting co-assembly phenomenon of a fuel pair (DiR and BNN6), which was discovered in pre-experimental attempts. Based on this exciting finding, we proposed exploiting a self-propelling nano-penetrator consisting of a DiR-BNN6 fuel pair with efficient NO generation and controllable motion features. To test our hypothesis, a photothermally activatable NO donor (BNN6) was synthesized starting from N, N’-bis(1-methylpropyl)−1,4-phenylenediamine (BPA) (Supplementary Fig. 1)^27^. The mass spectrometry, results confirmed the successful synthesis of BNN6 (Supplementary Fig. 2). Notably, BNN6 was unable to self-assemble into a stable nanostructure (Supplementary Fig. 3), but it could readily co-assemble with DiR into binary nanoassemblies (NAs) using a facile one-step nano-precipitation method without the assistance of any carrier materials. Significantly, BNN6 and DiR in the NAs could be precisely tailored to a wide range of molar ratios from 10:1 to 1:10 (Supplementary Tab. 1). The bare BNN6/DiR NAs without PEGylation or targeting modification was named as B-BD NAs (Fig. 2a, b). Given that laser-triggered NO generation from the carrier-free NAs depends heavily on the molar ratio of the BNN6-DiR fuel pair, the NO productivity and nanoassembly features of B-BD NAs was evaluated to determine the optimal combination ratio of the BNN6-DiR pair. As shown in Fig. 2c and Supplementary Tab. 1, B-BD NAs with a molar ratio of 1:3 (DiR/BNN6) were the optimal nanoassembly combination.

To improve the colloidal stability and thrombus-homing accumulation of B-BD NAs, DSPE-PEG_2K_ and DEPE-PEG_2K_-CREKA were utilized to fabricate PEGylated thrombus-targeting nanoassemblies (T-BD NAs)^28^. Moreover, the precursor (BPA) of BNN6 without NO production ability was used to prepare the control nanoassemblies (N-BD NAs). N-BD NAs were prepared similarly to T-BD NAs, except that BNN6 was replaced with BPA (Fig. 2b). As shown in Fig. 2d–f and Supplementary Tab. 2, B-BD NAs, T-BD NAs, and N-BD NAs showed regular spherical structures with uniform particle sizes ranging from 76 to 118 nm. The mean diameters of the NAs increased slightly after modification with DSPE-PEG_2K_ and DSPE-PEG_2K_-CREKA (Supplementary Tab. 2). Owing to the negatively charged phosphate group and peptide in DSPE-PEG_2K_ and DSPE-PEG_2K_-CREKA, the BNN6/DiR NAs displayed a positive-negative charge transformation from 18.33 to 7.65 mV (Supplementary Tab. 2). Negatively charged nanomedicines certainly benefit from long circulation in the blood by reducing the interactions with abundant plasma proteins^29,30^. As shown in Fig. 2g, T-BD NAs and N-BD NAs showed much better colloidal stability in PBS (pH 7.4) than non-PEGylated B-BD NAs under the same conditions. Moreover, B-BD NAs, T-BD NAs, and N-BD NAs showed excellent long-term stability at 4 °C under dark conditions, with no significant change in particle size (Supplementary Fig. 4). Subsequently, two artificial thrombus models (platelet-rich plasma and whole blood clots) were established to investigate the thrombus-targeting efficacy of NAs, respectively. As shown in Fig. 2h and Supplementary Fig. 5, T-BD NAs and N-BD NAs displayed much stronger fluorescence intensity in both platelet-rich plasma clots and whole blood clots than DiR Sol and B-BD NAs, suggesting the crucial role of CREKA in thrombus-homing accumulation. In addition, T-BD NAs showed high loading capacity of the fuel pair up to 41.1% and 33.9% for DiR and BNN6, respectively (Supplementary Tab. 2). That is, 75% of the components served as fuel in the nano-penetrator, which was much higher than any previously reported nanomaterial-based nanomotors. The high co-loading capacity of DiR and BNN6 in T-BD NAs certainly contributes to laser-triggered NO generation and durable motion, which is of great significance for the fuel pair-engineered nano-penetrator in the nonpharmaceutical treatment of thrombosis and ischemic stroke.

Furthermore, we focused on the nanoassembly mechanisms of the BNN6-DiR fuel pair. Molecular docking simulation technique was employed to analyze the intermolecular forces. As shown in Fig. 2i, multiple forces were identified in BNN6/DiR NAs, including hydrophobic force, electrostatic interaction, and π-π stacking interaction. Subsequently, sodium dodecyl sulfate (SDS), sodium chloride (NaCl), and urea were utilized as intermolecular interaction breakers to determine the primary assembly driving forces^31^. As shown in Fig. 2j, the mean diameters of B-BD NAs and T-BD NAs sharply increased in the presence of SDS and NaCl, suggesting the significant roles of hydrophobic and electrostatic interactions in the co-assembly process of BNN6 and DiR. In contrast, there was almost no change in particle size of the NAs incubated with urea under the same conditions, indicating the marginal contribution of hydrogen bonds to the formation of NAs. Moreover, BNN6 showed almost no UV absorption in the wavelength range from 400 to 900 nm, whereas the UV absorbance spectra of B-BD NAs and T-BD NAs showed an obvious red-shift when compared to DiR Sol (Fig. 2k), suggesting the existence of π-π stacking interaction in the fuel pair-engineered NAs^30^. Notably, the peak position in the fluorescence spectra of DiR did not change significantly before and after co-assembly with BNN6, while the fluorescence intensity of the NAs significantly decreased when compared to that of DiR Sol at the same concentration (Fig. 2l). The attenuated fluorescence intensity could be ascribed to the aggregation-induced quenching (ACQ) effect of DiR in NAs, which further confirmed the formation of BNN6/DiR co-assembly. Notably, the insertion of hydrophobic DSPE moiety in T-BD NAs contributed to a slight alleviation of the ACQ effect by increasing the molecular distance between DiR in the NAs (Fig. 2l).

### Laser-triggered NO generation and self‐fueled autonomous motion

The successful fabrication and favorable properties of T-BD NAs motivated us to further explore their photothermal conversion capacity, NO generation, and autonomous motion features under laser irradiation. As previously described, T-BD NAs were elaborately engineered based on the molecular co-assembly of a BNN6-DiR fuel pair, which is completely different from existing carrier materials-based nanomotors. In particular, DiR and BNN6 co-assembled side-by-side in the NAs acted as both the component elements and energy source of self-fueled nano-penetrator (Fig. 1). We expected that such a carrier-free photothermal nanomotor would inevitably facilitate laser-triggered NO generation and self-fueled autonomous motion under laser irradiation, owing to the efficient energy transfer between the BNN6-DiR fuel pair at a short molecular distance in the NAs.

**Fig. 3:**
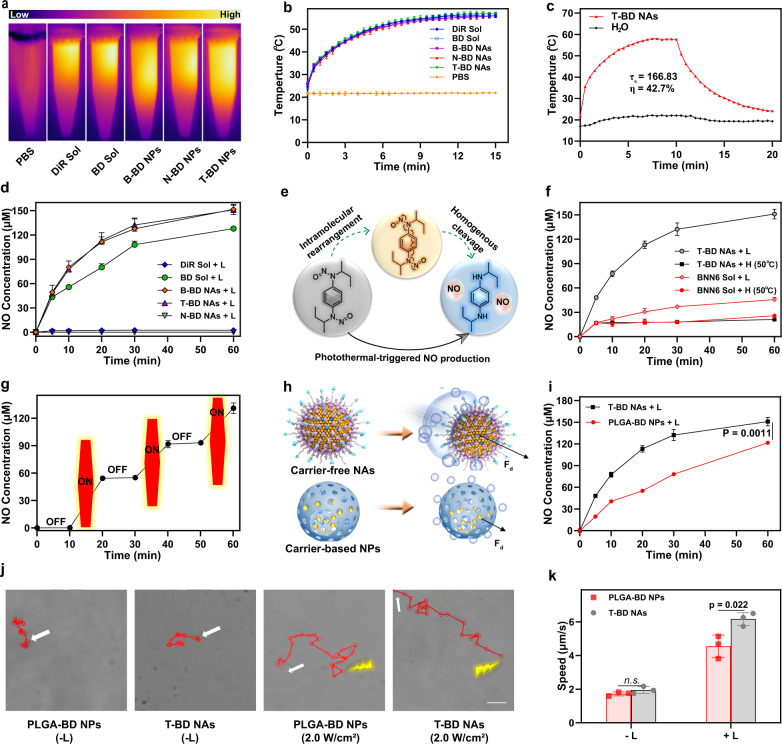
Laser-triggered NO generation and self-fueled autonomous motion. In vitro photothermal heating **a** image and **b** curves under laser irradiation (2 W/cm^2^). Data are presented as mean ± SD (*n* = 3 independent experiments). Source data are provided as a Source Data file. **c** In vitro photothermal conversion efficiency of T-BD NAs. **d** NO release curves under laser irradiation (2 W/cm^2^). Data are presented as mean ± SD (*n* = 3, independent experiments). Source data are provided as a Source Data file. **e** Chemical mechanisms of NO generation from BNN6: intramolecular rearrangement and homogenous cleavage of N-NO bonds. **f** NO release curves of BNN6 Sol and T-BD NAs under heating (50 °C) or laser irradiation (2 W/cm^2^). Data are presented as mean ± SD (*n* = 3, independent experiments). Source data are provided as a Source Data file. **g** Off-on NO generation variations by the switch of laser irradiation (2 W/cm^2^). Data are presented as mean ± SD (*n* = 3, independent experiments). Source data are provided as a Source Data file. **h** Schematic representation of NO generation and autonomous motion features of carrier-free NAs and carrier-based NPs. **i** Laser-triggered NO release from T-BD NAs and PLGA-BD NAs at the same dose of DiR/BNN6 (2 W/cm^2^). Data are presented as mean ± SD (*n* = 3, independent experiments). Source data are provided as a Source Data file. One-way ANOVA (one-sided) with Dunnett’s multiple comparisons test was used for the analysis of data and adjusted *P* value. **j** The movement trajectories of T-BD NAs and PLGA-BD NAs incubated in PBS (pH 7.4) at the same dose of DiR/BNN6 under laser irradiations (0 W/cm^2^ and 2 W/cm^2^, 60 s), scale bar = 1 μm. **k** The motion speed of T-BD NAs and PLGA-BD NPs incubated in PBS (pH 7.4) at the same dose of DiR/BNN6 under laser irradiations (2 W/cm^2^). Data are presented as mean ± SD (*n* = 3, independent experiments). Source data are provided as a Source Data file. One-way ANOVA (one-sided) with Dunnett’s multiple comparisons test was used for the analysis of data and adjusted P value. The n.s. represent no significance. Yellow lightning represents laser irradiation. *L* and *H* represent laser and heating, respectively.

To validate our design, we first explored the photothermal features of the fuel pair NAs under 808 nm laser irradiation (2 W/cm^2^, 15 min). As shown in Fig. 3a, b, the temperature of DiR Sol, BNN6/DiR mixture solution (BD Sol), B-BD NAs, N-BD NAs, and T-BD NAs continued to rise to >50 °C within 10 min under laser irradiation, whereas the temperature of PBS (pH 7.4) did not dramatically change under the same conditions. These results revealed that co-assembly with BNN6 and surface decoration on the NAs had no significant influence on the photothermal effect of DiR^32^. Moreover, the result showed that the photothermal conversion efficiency of T-BD NAs was up to 42.7% (Fig. 3c). The favorable photothermal efficiency of T-BD NAs would benefit NO generation and autonomous motion, and contribute to the deep penetration of NAs into the thrombi by virtue of local thermal effect.

The excellent photothermal properties of the BNN6/DiR NAs prompted us to evaluate their NO generation efficiency. The concentration of NO was quantitatively determined using the Griess assay (Supplementary Fig. 6)^33^. As shown in Fig. 3d, both B-BD NAs and T-BD NAs enabled abundant NO generation with no significant difference between them under 808 nm laser irradiation (2 W/cm^2^) within 60 min. These results indicated that PEGylation and CREKA peptide modifications had no impact on laser-triggered NO generation from BNN6/DiR NAs. Owing to the absence of the NO donor (BNN6), there was hardly any NO detected in N-BD NAs and DiR Sol under the same conditions (Fig. 3d). Notably, the amount of NO detected in BD Sol was significantly lower than that detected in B-BD NAs and T-BD NAs under the same conditions (Fig. 3d). This could be ascribed to the larger intramolecular distance between DiR and BNN6 in solution than that in the NAs, resulting in inefficient intermolecular energy transfer under laser irradiation.

We investigated the mechanism of NO production by BNN6 (Fig. 3e). As shown in Fig. 3f, only a minimal amount of NO was produced from BNN6 Sol and T-BD NAs when heated directly (50 °C), suggesting a modest effect of heat on NO production. In contrast, laser irradiation quickly triggered the abundant NO generation from T-BD NAs, along with on-going temperature rise up to ~50 °C (Fig. 3b, f). Notably, laser-triggered NO generation from T-BD NAs was significantly higher than that from BNN6 Sol under the same conditions (Fig. 3f), suggesting a significantly weakened thermal effect and energy transfer in the absence of DiR. The favorable NO productivity of T-BD NAs should be ascribed to a more efficient energy transfer from DiR molecules to the neighboring BNN6 molecules in the BNN6/DiR NAs under laser irradiation, which effectively triggered NO generation by promoting intramolecular rearrangement and initiating the homogenous cleavage of the N-NO bonds of BNN6 (Fig. 3e). These results suggested that the photothermal effect rather than heat effectively triggered NO generation from BNN6/DiR NAs. Moreover, laser-triggered NO production from T-BD NAs occurred in a concentration- and radiation-energy-dependent manner (Supplementary Figs. 7, 8). More interestingly, NO generation from T-BD NAs demonstrated regular off-on variations along with the laser irradiation switch, suggesting a controllable NO generation feature of the fuel pair-engineered NAs (Fig. 3g).

As previously mentioned, we expected that the carrier-free NAs of the BNN6-DiR fuel pair would have a distinct advantage over conventional nanocarrier-based nanomotors in terms of the fuel loading capacity, NO generation and autonomous motion (Fig. 3h). To test our hypothesis, a commonly used PLGA nanocarrier was prepared to co-encapsulate the same fuel pair (DiR/BNN6, 1:3), with a low fuel loading rate of 6.25% (DiR/BNN6). It was named PLGA-BD NPs (Supplementary Fig. 9). We then compared the photothermal properties of T-BD NAs and PLGA-BD NPs under laser irradiation (2 W/cm^2^, 10 min). As shown in Fig. 3i and Supplementary Fig. 10, T-BD NAs showed comparable photothermal conversion efficiency with PLGA-BD NPs at the same DiR dose, whereas T-BD NAs revealed much more efficient NO production than PLGA-BD NPs under the same conditions. Although the photothermal conversion efficiency was comparable to that of carrier-free T-BD NAs, the energy transfer between DiR and BNN6 entrapped in PLGA-BD NPs was significantly compromised by the heavy use of PLGA polymers, resulting in inferior NO productivity of carrier material-based nanomotors (Fig. 3h).

Subsequently, we comparatively investigated the NO-driven autonomous motion behaviors of T-BD NAs and PLGA-BD NPs under 808 nm laser irradiation (0-2 W/cm^2^, 60 s). Movement trajectories were recorded using a fluorescence microscope with Nikon camera under short-time laser irradiation. As shown in Fig. 3j, k, Supplementary Fig. 11–12 and Supplementary Movie 1–2, T-BD NAs swam vigorously in PBS (pH 7.4) in a laser power-dependent manner, while PLGA-BD NPs showed inferior autonomous motion ability under the same conditions. Notably, the NO-driven autonomous motion trajectory accurately reflected the motor feature of a single particle. As expected, carrier-free T-BD NAs showed distinct advantages over carrier-based PLGA-BD NPs in terms of fuel loading capacity, energy transfer efficiency, NO productivity, and autonomous motion. Moreover, in order to explore the autonomous motion ability of T-BD NAs in a simulated physiological environment, T-BD NAs were incubated with PBS (pH 7.4) containing 10% FBS and imposed short-time 808 nm laser irradiation (2 W/cm^2^, 60 s). As shown in Supplementary Fig. 13, T-BD NAs still displayed favorable autonomous motion ability in PBS (pH 7.4) containing 10% FBS under laser irradiation. These results confirmed our hypothesis that the carrier-free NAs of fuel pair with excellent autonomous motion ability would provide a promising self-propelled nano-penetrator for biomedical applications. More importantly, the carrier-free nanomotor consisting of a photothermal fuel pair demonstrated precisely laser-triggered autonomous motion features, particularly compared to those nanomotors consisting of endogenous stimuli-activatable NO donors^18^. NO generation from the latter could inevitably be affected by the heterogeneity of pathological microenvironments.

### In vitro synergistic thrombolysis and clot-deep penetration

**Fig. 4:**
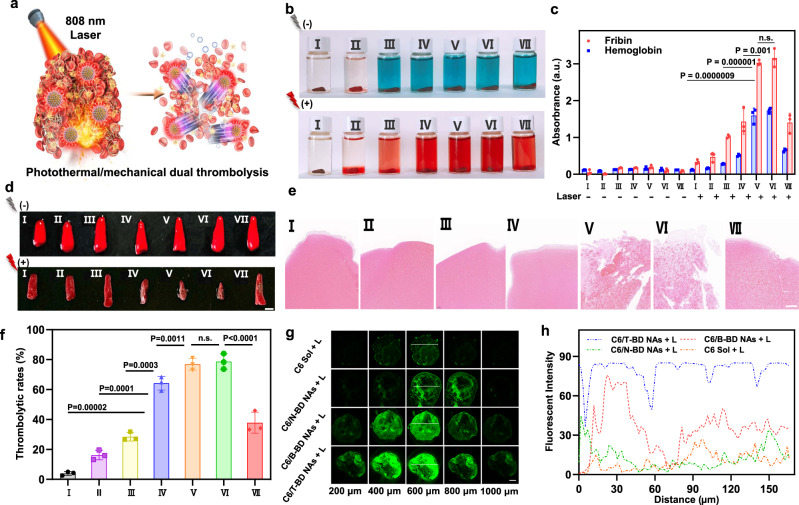
In vitro synergistic thrombolysis and clot deep penetration. **a** Schematic representation of photothermal/mechanical synergistic thrombolysis and clot-deep penetration. **b** Photos of blood clots after different treatments with/without the 808 nm NIR laser irradiation: (2 W/cm^2^, 20 min). **c** Fibrin and hemoglobin levels in the blood clot supernatants after different treatments with/without the 808 nm NIR laser irradiation. Data are presented as mean ± SD (*n* = 3, independent experiments). Source data are provided as a Source Data file. One-way ANOVA (one-sided) with Dunnett’s multiple comparisons test was used for the analysis of data and adjusted *P* value. The n.s. represent no significance. **d** Images of blood clots before and after different treatments with 808 nm NIR laser irradiation (2 W/cm^2^, 20 min), scale bar = 2 mm. **e** H&E staining results of **d** under 808 nm NIR laser irradiation (2 W/cm^2^, 20 min), scale bar = 100 μm. **f** Thrombolytic rates of **d** under 808 nm NIR laser irradiation (2 W/cm^2^, 20 min). Data are presented as mean ± SD (*n* = 3, independent experiments). Source data are provided as a Source Data file. One-way ANOVA (one-sided) with Dunnett’s multiple comparisons test was used for the analysis of data and adjusted *P* value. The n.s. represent no significance. **g** CLSM images of small red thrombi incubated with free C-6, C6/B-BD NAs, C6/N-BD NAs, or C6/T-BD NAs under 808 nm laser irradiation (2 W/cm^2^, 15 min), scale bar = 100 μm. Experiment was repeated three times independently with similar results. **h** Quantitative results of the depth of clot penetration. (I: PBS, II: BNN6 Sol, III: DiR Sol, IV: BD Sol, V: B-BD NAs, VI: T-BD NAs, VII: N-BD NAs). The lightning bolt represents laser irradiation.

As previously mentioned, we expected that the fuel pair-engineered nano assembly of a photothermal photosensitizer (DiR) and a photothermal-activable NO donor (BNN6) could serve as a versatile nano-penetrator for the nonpharmaceutical treatment of thrombotic diseases. The in vitro photothermal/mechanical synergistic thrombolysis and clot deep penetration were studied (Fig. 4a). First, the in vitro thrombolytic effects were evaluated by determining the thrombolytic rates and the UV absorption of fibrin and hemoglobin derived from the clots^15^. As illustrated in Fig. 4b, c, blood clots remained intact at the bottom of bottles with very little fibrin and hemoglobin detected in the supernatants^34^. Notably, the color of supernatants came from DiR without laser irradiation. As shown in Fig. 4b–f, DiR Sol, BD Sol, and N-BD NAs showed moderate thrombolysis effects under laser irradiation (2 W/cm^2^, 20 min), owing to the limited therapeutic efficiency by photothermal thrombolysis alone. Notably, both BD NAs and T-BD NAs exhibited favorable synergistic thrombolysis effect, with significantly increased fibrin and hemoglobin levels in the supernatants and significantly decreased volume of blood clots after laser irradiation (Fig. 4b–f). The favorable outcomes of B-BD NAs and T-BD NAs should be ascribed to a collaborative result of DiR-based photothermal thrombolysis and NO-mediated mechanical thrombolysis (Fig. 4d). As a result, the combined photothermal/mechanical thrombolytic effect of B-BD NAs and T-BD NAs showed distinct advantage over N-BD NAs without autonomous motion ability. Moreover, PEGylation and targeting modifications on the fuel pair NAs had little impact on in vitro thrombolysis effects. Taken together, these results suggested that the molecularly engineered NAs of BNN6-DiR fuel pair can act as a self-propelled nano-penetrator for nonpharmaceutical thrombolysis in a photothermal/mechanical synergistic manner (Fig. 4f).

The local hyperthermal effect of PTT has been found to effectively promote deep penetration of nanomedicines into thrombi^35,36^. Meanwhile, gas-driven nanomotors can also effectively penetrate biological barriers through mechanical motion^37^. We proposed that the combination of hyperthermal effect and mechanical motion would further facilitate the deep clot penetration of NAs. A small red thrombus model was constructed to evaluate the penetration and retention features of the BNN6/DiR NAs. Coumarin-6 (C6) was utilized to label the NAs (C6/B-BD NAs, C6/N-BD NAs, and C6/T-BD NAs). C6 Sol was utilized as the control. As shown in Fig. 4g, h and Supplementary Fig. 14, C6/N-BD NAs without NO generation capacity showed moderate photothermal-promoted penetration effect, but better than that of C6 Sol under the same conditions (Fig. 4g, h, Supplementary Fig. 14). In contrast, both C6/T-BD NAs and C6/B-BD NAs demonstrated much stronger fluorescent signals in the deep regions of the thrombi than other groups under 808 nm laser irradiation (2 W/cm^2^, 15 min). Moreover, C6/T-BD NAs showed an even deeper penetration distance than C6/B-BD NAs under the same conditions (Fig. 4g, h, Supplementary Fig. 14), which could probably be ascribed to the targeting action of CREKA peptide modified on C6/T-BD NAs. These results confirmed our hypothesis that self-propelled nano-penetrator with excellent photothermal/mechanical clot deep penetration could serve as a novel nanotherapeutics for nonpharmaceutical treatment against thrombotic diseases.

### Pharmacokinetics and thrombus-specific biodistribution

**Fig. 5:**
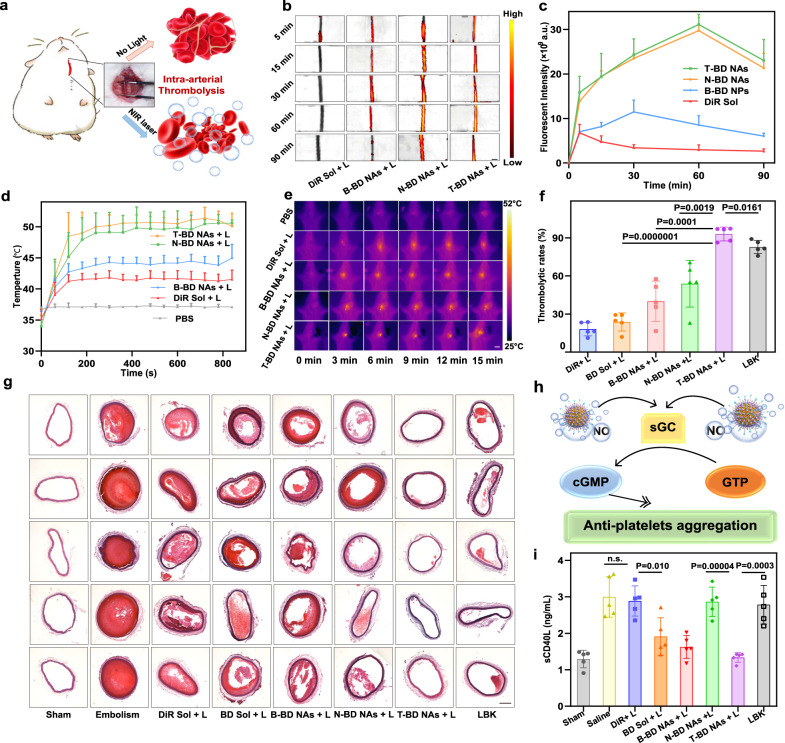
Intra-arterial thrombolysis in SD rats (*n* = 5). **a** Schematic representation of photothermal/mechanical synergistic thrombolysis effects in a FeCl_3_-induced rat carotid arterial thrombosis model. **b** Representative fluorescence images (scale bar = 2 mm) and **c** quantitative results of the carotid artery embolic vessels after intravenous injection of DiR Sol, B-BD NAs, T-BD NAs, or N-BD NAs at an equivalent DiR dose of 5 mg/kg. Data are presented as mean ± SD. Source data are provided as a Source Data file. In vivo photothermal heating curves **d** and images **e** of carotid artery thrombotic vessels under 808 nm laser irradiation (2.0 W/cm^2^, 15 min), scale bar = 2 cm. Data are presented as mean ± SD. Source data are provided as a Source Data file. **f** Nonpharmaceutical thrombolysis rates of carotid arterial vessel sections after various treatments, quantitatively analyzed by software Image-pro plus. Data are presented as mean ± SD. Source data are provided as a Source Data file. One-way ANOVA (one-sided) with Dunnett’s multiple comparisons test was used for the analysis of data and adjusted *P* value. **g** Nonpharmaceutical thrombolysis results of carotid arterial vessels sections after various treatments, scale bar = 100 μm. **h** Schematic diagram of NO-mediated thrombolysis mechanism: antiplatelet aggregation. **i** The sCD40L levels in the blood samples of rats after various treatments. Data are presented as mean ± SD. Source data are provided as a Source Data file. One-way ANOVA (one-sided) with Dunnett’s multiple comparisons test was used for the analysis of data and adjusted *P* value. The n.s. represent no significance.

As previously mentioned, Janus nanomotors with asymmetric nanostructures could be rapidly phagocytized by the RES and quickly cleared from the blood circulation. The poor pharmacokinetic profiles of Janus NPs significantly impeded their biomedical applications. In contrast, spherical-shaped NPs with suitable hydrophilic modifications (e.g., PEGylation) have been characterized by extended circulation features in the blood after intravenous injection. We expected that the self-fueled nano-penetrator (T-BD NAs) with spherical morphology and hydrophilic PEGylation modification would exhibit favorable pharmacokinetics after intravenous injection. Moreover, decoration with CREKA peptide would certainly facilitate site-specific accumulation of the nano-penetrator in thrombi. In this part, healthy Sprague-Dawley (SD) rats and FeCl_3_-induced arterial thrombosis SD rat model were utilized to explore the pharmacokinetics and thrombus-specific biodistribution of BNN6/DiR NAs, respectively (Fig. 5a–c, Supplementary Fig. 15).

The pharmacokinetic profiles of DiR Sol, B-BD NAs, N-BD NAs, and T-BD NAs were investigated in SD rats. The plasma concentrations of DiR were determined by fluorescence analysis at different time points. As shown in Supplementary Fig. 15 and Supplementary Tab. 3, the fluorescence signals of DiR Sol group were quickly attenuated after intravenous injection, suggesting the inferior pharmacokinetics of free drug solution. Moreover, although it exhibited a slightly better pharmacokinetic profile than DiR Sol, the non-PEGylated B-BD NAs were still quickly cleared from the blood circulation (Supplementary Fig. 15), which should be ascribed to its inferior colloidal stability (Fig. 2g). In contrast, both T-BD NAs and N-BD NAs showed much longer circulation time in the blood than the DiR Sol and B-BD NAs (Supplementary Fig. 15). PEGylation modification on the fuel pair-engineered NAs not only greatly improved colloidal stability in vitro (Fig. 2k), but also significantly extended the systemic circulation time in vivo (Supplementary Fig. 15).

By virtue of the CREKA peptide decoration and the fluorescence feature of DiR, T-BD NAs were expected to enable self-navigating and self-indicating thrombus-specific delivery. We subsequently investigated the thrombus-homing accumulation of DiR Sol and BNN6/DiR NAs in a FeCl_3_-induced arterial thrombosis rat model. As shown in Fig. 5b, c, both DiR Sol and B-BD NAs showed fragile fluorescence signals in the obstructed vessels within 120 min, which should be attributed to poor pharmacokinetics and lack of CREKA-mediated targeting ability. In contrast, both N-BD NAs and T-BD NAs displayed much stronger fluorescence intensity in the obstructed vessels than DiR Sol and B-BD NAs (Fig. 5b). These results indicated the excellent self-navigating and self-indicating thrombus-specific delivery feature of BNN6/DiR NAs with PEGylation and targeting modification, which would certainly benefit precise intervention on thrombotic diseases in vivo. Notably, the fluorescence signals in the blood clots increased first and then decreased after intravenous injection with the NAs (Fig. 5c). The strongest fluorescence intensity of N-BD NAs and T-BD NAs was observed at 60 min post-injection (Fig. 5c), which contributed to figuring out an optimal irradiation time in the therapeutic schedule in vivo.

### Intra-arterial thrombolysis

The outstanding nanoassembly feature, photothermal conversion, NO productivity, autonomous motion, in vitro thrombolytic efficacy, pharmacokinetics, and the self-navigated thrombus-targeting capacity of T-BD NAs make it a promising self-fueled nano-penetrator for nonpharmaceutical treatment against thrombotic diseases. We first investigated the in vivo photothermal conversion efficiency in FeCl_3_-induced rat carotid arterial thrombosis model. Under 808 nm laser irradiation, the temperatures of the obstructed vessels treated with PBS, DiR Sol, B-BD NAs, N-BD NAs, and T-BD NAs were monitored using a thermal infrared camera, respectively. As shown in Fig. 5d, e, the clot temperature of the rats receiving N-BD NAs and T-BD NAs rapidly increased up to ~51 °C after irradiation with 808 nm laser for 15 min (2 W/cm^2^), which was much higher than that of B-BD NAs (~42 °C) and DiR Sol (~40 °C). Notably, despite the comparable in vitro photothermal properties of the non-PEGylated B-BD NAs and DiR Sol (Fig. 3a, b), N-BD NAs and T-BD NAs with PEGylation and targeting modification displayed distinct advantages in terms of in vivo photothermal conversion, which should be ascribed to favorable pharmacokinetic profiles (Supplementary Fig. 15) and CREKA peptide-driven thrombus-targeting delivery (Fig. 5b, c).

Subsequently, the photothermal/mechanical thrombolysis efficacy of the nano-penetrator was evaluated in FeCl_3_-induced rat carotid arterial thrombosis model. As shown in Fig. 5f, g, DiR Sol, BD Sol, and B-BD NAs with poor pharmacokinetic profiles and inferior thrombus-targeting ability displayed inferior thrombolysis activity under 808 nm laser irradiation (2 W/cm^2^, 10 min). Notably, despite the favorable pharmacokinetics, thrombus-targeting feature, and photothermal conversion of the control N-BD NAs without NO production ability, only moderate thrombolysis was demonstrated under the same conditions (Fig. 5f, g), suggesting the inadequate effect of photothermal thrombolysis alone. As expected, T-BD NAs showed potent antithrombotic activity with a high thrombolytic rate (~90%) under 808 nm laser irradiation (Fig. 5f), suggesting excellent photothermal/mechanical synergistic thrombolysis efficacy of the fuel pair-engineered nano-penetrator. Notably, T-BD NAs showed even stronger thrombolytic activity than lumbrokinase (LBK, a clinical first-line thrombolytic drug), which further verified the favorable nonpharmaceutical thrombolysis ability of the fuel pair nano-penetrator (Fig. 5f, g). Moreover, as shown in Fig. 5h, it has been found that NO could effectively inhibit platelet aggregation by transforming guanosine triphosphate into cyclic guanosine monophosphate (cGMP)^38^. Therefore, we further evaluated the antiplatelet activity of T-BD NAs by measuring the expression of sCD40L in the blood, which would be highly expressed once platelet activation. As shown in Fig. 5i, the levels of sCD40L were significantly elevated after carotid thrombosis by FeCl_3_ compared to the Sham group. Notably, BD Sol, B-BD NAs, and T-BD NAs could effectively downregulate the sCD40L levels under laser irradiation, suggesting the potent antiplatelet activity of the formulations with NO generation ability. Subsequently, we further evaluated the anticoagulation activity of T-BD NAs by measuring the levels of activated partial thromboplastin time (APTT) after different treatments. As shown in Supplementary Fig. 16, the Embolism group exhibited significant APTT reduction when compared to the Sham group, suggesting the formation of thrombus. Notably, the APTT levels in DiR Sol, N-BD NAs, and LBK groups exhibited no significant difference with the Embolism group (Supplementary Fig. 16), whereas BD Sol and B-BD NAs effectively inhibited the activation of prothrombin with remarkable increase in APTT levels (Supplementary Fig. 16). Notably, T-BD NAs presented the strongest anticoagulation activity with the highest APTT level among all these formulations. The anticoagulation activities of BD Sol, B-BD NAs, and T-BD NAs should be ascribed to laser-triggered NO generation. The potent in vivo anticoagulation activity of T-BD NAs benefited from its excellent pharmacokinetic behavior with long circulation time in the blood (Supplementary Fig. 15). Taken together, the mechanical thrombolysis/mechanical thrombolysis and NO-mediated platelet inhibition of nano-penetrator resulted in favorable antithrombotic efficacy in vivo.

Furthermore, thrombolytic fragments produced after thrombolysis therapy might induce secondary thrombosis in the small vessels (e.g., pulmonary embolism), which has been regarded as a serious challenge for clinical arterial thrombosis therapy^39,40^. To check for any blockage lesions, the major organs (heart, liver, spleen, lung, and kidney) were collected from the rats after treatment with T-BD NAs for 14 days. As shown in Supplementary Fig. 17, T-BD NAs did not induce any pulmonary embolism in the lung, and other primary organs (heart, liver, spleen, and kidney). The favorable antithrombotic effect without tissue damage should be attributed to the anticoagulant and antiplatelet effects of NO generated from the nano-penetrator (Fig. 5h, i, Supplementary Fig. 16). Moreover, even if there were some small pieces of clots produced during the therapeutic process, most of them could be degraded into very small fragments by blood flow, and eventually filtered through the anticlogging system of glomerular filtration membranes^41^.

Moreover, we further explored the potential vasculature damage during treatment with T-BD NAs. The H&E staining sections of carotid artery embolism blood vessels before and after treatment with T-BD NAs under laser irradiation (808 nm) were investigated. Notably, Embolism group and T-BD NAs + L both exhibited faint damage to the nearby blood vessels when compared to the Sham group (Supplementary Fig. 18). Such a faint vascular damage may ascribe to the FeCl_3_ used for modeling rather than short-term laser irradiation, suggesting favorable therapeutic safety in vivo. The good therapeutic safety of T-BD NAs should be attributed to fibrin-targeting modification, which facilitated the specific accumulation of NAs in thrombi instead of vascular walls. Moreover, temperature rise in thrombi contributed to loosening the clots by breaking the non-covalent bonding between fibrins rather than “burning” the clots, which not only exerted photothermal thrombolysis but also did not cause obvious damage to the nearby blood vessels^9^.

### Intravenous thrombolysis

**Fig. 6:**
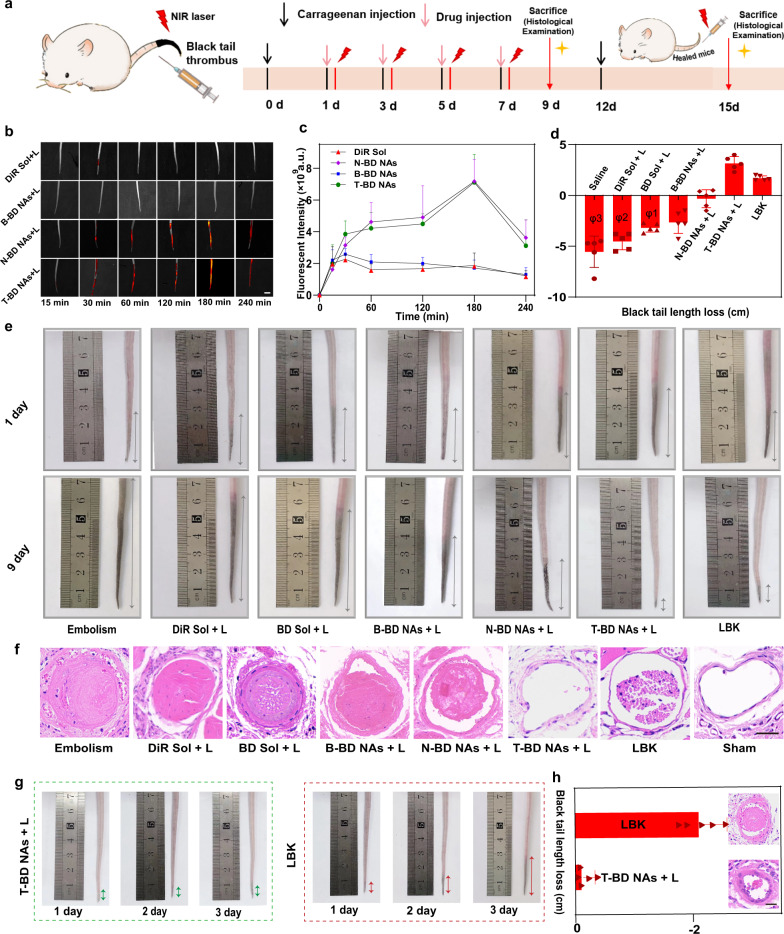
Intravenous thrombolysis and prevention recurrence of thrombus (*n* = 5). **a** Treatment scheme in intravenous thrombolysis therapy. **b** Representative fluorescence images (scale bar = 1 cm) and **c** quantitative results of the tail thrombus model mice after different treatments (DiR equivalent dose 5 mg/kg). Data are presented as mean ± SD. Source data are provided as a Source Data file. **d** The average black tail length loss of the tail thrombus model mice before and after 9 days with the injection of different formulations with or without laser irradiation (DiR equivalent dose 5 mg/kg, *φ* represents the amputated tail). Data are presented as mean ± SD. Source data are provided as a Source Data file. **e** Photos of black tails before and after different treatments. **f** Representative thrombolysis results of tail thrombus slices from the different groups of **e**, scale bar = 100 µm. **g** Photographs of the thrombus recurrence progress after treatment with T-BD NAs + *L* (2 W/cm^2^, 15 min) or LBK. **h** Average black tail length loss of the tail thrombus model mice and representative H&E-stained images of tail thrombus slices after treatment with T-BD NAs + *L* (2 W/cm^2^, 15 min) or LBK (DiR equivalent dose 5 mg/kg), scale bar = 100 μm. Data are presented as mean ± SD. Source data are provided as a Source Data file.

In addition to arterial thrombus, we also expected that the fuel pair-engineered nano-penetrator would exert favorable therapeutic effects against venous thrombus. Kunming (KM) mice tail thrombosis model was established by intraperitoneal injection of carrageenan (Fig. 6a). The site-specific accumulation and temperature elevation of DiR Sol, B-BD NAs, N-BD NAs, and T-BD NAs in thrombi was monitored. As shown in Fig. 6b, c, N-BD NAs, and T-BD NAs displayed much stronger fluorescence intensity in the obstructed tail vessels than that of DiR Sol and B-BD NAs, which were consistent with the results in the FeCl_3_-induced rat carotid arterial thrombosis model (Fig. 5b, c). Similarly, the favorable thrombus-homing accumulation of N-BD NAs and T-BD NAs should be attributed to long systemic circulation time and CREKA peptide-mediated targeting delivery. Moreover, the fluorescence signals of N-BD NAs and T-BD NAs first raised then fell with a peak fluorescence intensity at 3 h post-injection (Fig. 6c). As shown in Supplementary Fig. 19, thrombus local temperature of the mice injected with T-BD NAs rapidly increased up to ~47 °C under 808 nm laser irradiation at 3 h post-injection (2 W/cm^2^, 15 min). These results suggested the excellent thrombus-homing distribution and photothermal properties of the fuel pair-engineered nano-penetrator in the venous thrombus.

We then evaluated the nonpharmaceutical thrombolysis of T-BD NAs in the KM mice tail thrombosis model by comparing the length of black tails. As shown in Fig. 6d, e, black tail length of the mice injected with saline, DiR Sol and BD Sol significantly increased under 808 nm laser irradiation (2 W/cm^2^, 15 min). Owing to the inferior thrombolysis effects, high probability of tail amputation events occurred in the Embolism groups, DiR Sol and BD Sol (Fig. 6d). Tail amputation cases were labeled by *φ*, which refers to the number of amputated tails in the treatment process (Fig. 6d). Moreover, the black tail length of mice receiving naked B-BD NAs or non-gas N-BD NAs also increased to a certain extent under the same conditions (Fig. 6d). In contrast, the black tails of mice receiving fuel pair nano-penetrator (T-BD NAs) gradually healed without the occurrence of tail amputation case under 808 nm laser irradiation (Fig. 6d, e), suggesting excellent nonpharmaceutical thrombolysis against venous thrombus. Meanwhile, T-BD NAs revealed more potent intravenous thrombolysis activity than LBK (Fig. 6d, e), which was consistent with the histological analysis results (Fig. 6f).

As previously mentioned, there is increasing evidence that NO exerts anticoagulation and antiplatelet effects through increasing cyclic GMP and downregulating platelet aggregation. So, we next explored the anticoagulation activity of nano-penetrator by evaluating the APTT levels in the blood. As shown in Supplementary Fig. 20, the APTT in Embolism group was drastically reduced when compared to the Sham group. Moreover, the NO-producing groups (BD Sol, B-BD NAs, and T-BD NAs) significantly prolonged the APTT in mouse tail thrombosis model (Supplementary Fig. 20), which should be ascribed to the important roles of NO in inhibiting platelet adhesion and blood coagulation^38^. Among them, T-BD NAs showed a more potent anticoagulation effect than that of BD Sol and B-BD NAs, owing to the favorable pharmacokinetic profiles and thrombus-targeting ability of fuel pair nano-penetrator. Notably, LBK had little impact on the APTT, since it primarily acts on fibrinogen rather than prothrombin in the blood^42,43^. These results were consistent with the therapeutic outcomes in FeCl_3_-induced rat carotid arterial thrombosis model (Fig. 5h, i and Supplementary Fig. 16). NO-mediated anticoagulation and antiplatelet effects would certainly benefit the prevention recurrence of thrombosis.

### Prevention recurrence of thrombus

It has been widely recognized that timely thrombolysis and prevention recurrence are equally important for the eradication of thrombotic diseases. Inspired by potent anticoagulation and antiplatelet effect of T-BD NAs, we supposed that vast amounts of NO generated from T-BD NAs under laser irradiation would not only severe as the driving force for mechanical thrombolysis, but also act as an important biological mediator for thrombus recurrence prevention. In this section, we evaluated the preventing effect of nano-penetrator in KM mice tail thrombosis model. As previously described, the mice tail thrombosis model was established by intraperitoneal injection of carrageenan. LBK was utilized as the positive control. The mice were intraperitoneally injected with carrageenan again at 12 d post treatment with T-BD NAs or LBK to induce the recurrence of thrombus. As shown in Fig. 6g, h, noticeable black tail events occurred in the LBK group on the third-day post carrageenan injection, while the mice receiving T-BD NAs displayed negligible recurrence under the same conditions. Moreover, the histological analysis results also confirmed the outstanding performance of T-BD NAs in preventing secondary thrombus formation, which should be attributed to a collaborative result of NO-mediated nonpharmaceutical thrombolysis and thrombus recurrence prevention. These results suggested that the molecularly self-fueled nano-penetrator could be used as a versatile nonpharmaceutical modality for thrombolytic therapy and prevention of thrombus recurrence.

### Ischemic stroke salvage

Thrombotic cerebrovascular diseases such as ischemic stroke have been considered the leading causes of death^44^. Notably, the narrow therapeutic window for acute ischemic stroke is usually <5 h^45,46^. Therefore, it is crucial to simultaneously perform thrombolysis and ischemic stroke salvage. To the best of our knowledge, there is no clinical therapeutics exerting both thrombolysis and neuroprotection in one dose. The above results have already indicated that the molecularly self-fueled nano-penetrator (T-BD NAs) not only demonstrated potent photothermal/mechanical thrombolysis activity, but also displayed great potential in preventing thrombus. More importantly, it revealed distinct therapeutic advantages over the clinical thrombolytic drug LBK. Given the beneficial effects of NO on stroke lesions, vast amounts of NO generated from the nano-penetrator under laser irradiation would represent a promising avenue for ischemic stroke relief^39^.

**Fig. 7:**
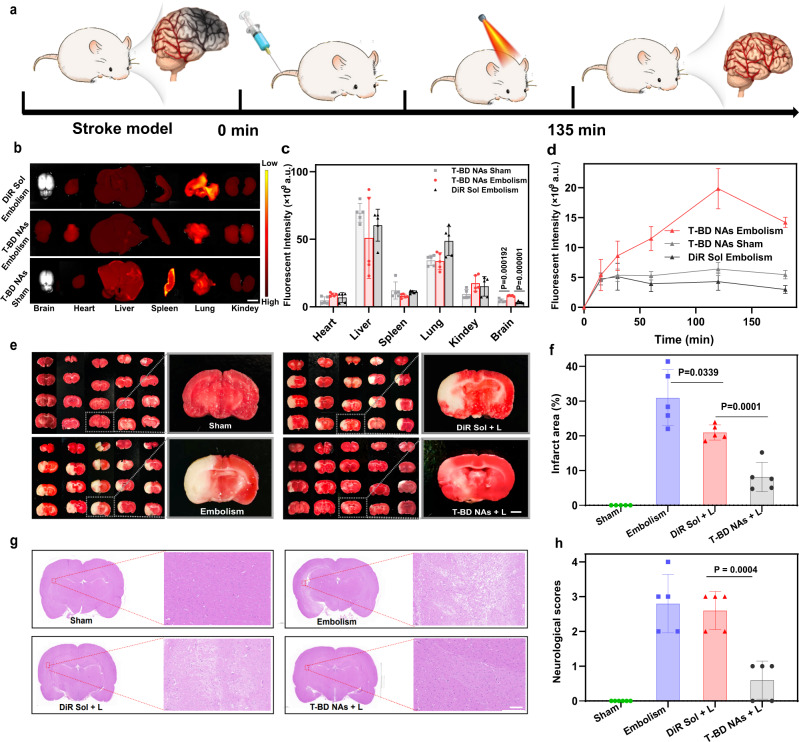
Ischemic stroke salvage (*n* = 5). **a** Treatment scheme in ischemic stroke salvage. **b** Representative tissue fluorescence imaging images of the MCAO model rats (Embolism) and health rats (Sham) after intravenous injection of T-BD NAs and DiR Sol, scale bar = 1 cm. **c** Fluorescence quantitative results of major organs in the MCAO model rats (Embolism) and health rats (Sham). Data are presented as mean ± SD. Source data are provided as a Source Data file. One-way ANOVA (one-sided) with Dunnett’s multiple comparisons test was used for the analysis of data and adjusted *P* value. **d** Brain fluorescence quantitative results of T-BD NAs and DiR Sol in the MCAO model rats (Embolism) and health rats (Sham) after intravenous injection. Data are presented as mean ± SD. Source data are provided as a Source Data file. **e** Representative photographs of TTC-stained brain slices of Sham group, Embolism group (0.9% NaCl), DiR Sol + L group (2 W/cm^2^, 15 min), and T-BD NAs + L group (2 W/cm^2^, 15 min), the infarct area is observed as white, scale bar = 1 mm. **f** Brain infarct volumes of the Sham group, Embolism group (0.9% NaCl), DiR Sol + L group (2 W/cm^2^, 15 min) and T-BD NAs + L group (2 W/cm^2^, 15 min), respectively. Data are presented as mean ± SD. Source data are provided as a Source Data file. One-way ANOVA (one-sided) with Dunnett’s multiple comparisons test was used for the analysis of data and adjusted *P* value. **g** Representative H&E staining results of the brain tissues in the Sham group, Embolism group (0.9% NaCl), DiR Sol + L group (2 W/cm^2^, 15 min), and T-BD NAs + L group (2 W/cm^2^, 15 min), respectively. **h** Neurological scores of the rats in the Sham group, Embolism group (0.9% NaCl), DiR Sol + L group (2 W/cm^2^, 15 min), and T-BD NAs + L group (2 W/cm^2^, 15 min), respectively. Data are presented as mean ± SD. Source data are provided as a Source Data file. One-way ANOVA (one-sided) with Dunnett’s multiple comparisons test was used for the analysis of data and adjusted *P* value.

We examined the therapeutic effect of T-BD NAs in ischemic stroke relief (Fig. 7a). A rat cerebral ischemia/reperfusion injury model was established by using the middle cerebral artery occlusion (MCAO). Briefly, SD rats were randomly divided into four groups (Sham, Embolism, DiR Sol + L, and T-BD NAs + L). First, the biodistribution of DiR Sol and T-BD NAs was investigated in the brains of healthy rats (Sham) and MCAO rats (Embolism), respectively. After the MCAO surgery, DiR Sol and T-BD NAs were intravenously injected into the MCAO rats, respectively. Meanwhile, an equivalent dose of T-BD NAs was intravenously injected into the healthy rats. Then, the brain-specific accumulation of T-BD NAs was analyzed utilizing the IVIS Spectrum imaging platform. As shown in Fig. 7b–d and Supplementary Fig. 20, the MCAO rats injected with T-BD NAs showed much stronger fluorescence signals in the brain tissues than that of the rats receiving DiR Sol, which should be ascribed to the long blood circulation time of T-BD NAs (Supplementary Fig. 15). Notably, the MCAO rats injected with T-BD NAs also showed much stronger fluorescence signals in the brain than that of healthy rats (Sham). There is growing evidence showing that ischemic stroke (IS) injury and ischemia-reperfusion injury could induce failure of BBB by increasing paracellular permeability post reperfusion, thereby significantly enhancing the permeability of T-BD NAs through the BBB. Notably, the strongest fluorescence intensity of DiR Sol and T-BD NAs in the brain of MCAO rats was observed at 60 min and 120 min post-injection, respectively (Supplementary Fig. 21), which provided the basis for laser irradiation time for ischemic stroke salvage.

We then evaluated the ischemic stroke relief of T-BD NAs in the rat cerebral ischemia/reperfusion injury model. After treatment with DiR Sol or T-BD NAs under laser irradiation (2 W/cm^2^, 15 min), the brains were harvested and stained by triphenyl tetrazolium chloride to analyze the area of cerebral infarction. Moreover, the neurological deficit scores of rats were evaluated. As shown in Fig. 7e, f, there were significant neurological deficits and infarcts in DiR-treated group and the MCAO embolism group, but none in the sham group. Obviously, DiR-based PTT alone was unbaled to effectively alleviate ischemic stroke. In contrast, T-BD NAs dramatically reduced the ischemic areas with an infarct rate down to ~5% (Fig. 7f), which showed a distinct advantage over the existing strategies with infarct rates of 10–20% reported in most literatures^42^. Moreover, T-BD NAs not only significantly relieved ischemic stroke in a rat cerebral ischemia/reperfusion injury model (Fig. 7g), but also did not cause significant damage to brain tissues (Supplementary Fig. 22). Moreover, the nerve function defect integral was found to significantly decrease in the rats receiving T-BD NAs (Fig. 7h). The outstanding performance of T-BD NAs in ischemic stroke treatment should be attributed to its multiple advantages, including high fuel loading capacity, efficient laser-triggerable NO generation, long circulation time in the blood, favorable distribution in the brains, as well as NO-facilitated vasodilation, neuroprotection and infarct tissue repair. These results indicated that the fuel pair-engineered nano-penetrator had great potential to serve as a versatile nonpharmaceutical modality for a closed-loop treatment of thrombosis and thrombotic diseases.

### Safety evaluation

We evaluated the in vitro and in vivo safety of T-BD NAs. First, hemolysis assay was performed to investigate the effect of T-BD NAs on red blood cells (RBCs). Briefly, the RBCs were collected from rats and incubated with PBS, DiR Sol, BNN6 Sol, BD Sol, B-BD NAs, T-BD NAs or N-BD NAs over time with/without laser irradiation, respectively. Pure water was utilized as a positive control. As shown in Supplementary Fig. 23, none of these formulations caused hemolysis with/without laser irradiation, suggesting the good biocompatibility of BNN6/DiR NAs for intravenous injection application. Subsequently, the potential cytotoxicity of DiR Sol, BNN6 Sol, BD Sol, B-BD NAs, T-BD NAs, and N-BD NAs was further investigated on human umbilical vein endothelial cells (HUVEC) with/without laser irradiation. As shown in Supplementary Fig. 24, all these formulations showed no significant cytotoxicity on HUVEC with/without laser irradiation (2 W/cm^2^, 5 min) at a DiR concentration range of 0–200 μM. These results suggested the good safety of T-BD NAs in vascular endothelial tissues.

Additionally, the hepatic and renal toxicity of T-BD NAs was evaluated at 24 h post intravenous injection. The biochemical indexes of serum, liver, and kidney function were measured, including ALT, AST, BUN, and creatinine levels. Moreover, the major tissues (heart, liver, spleen, lung, and kidney) were collected to stain by H&E and observed using a microscope. As shown in Supplementary Figs. 25, 26, T-BD NAs did not induce any variation in the biochemical indexes of serum and exhibited negligible histological damages in the major tissues (Supplementary Fig. 26). Taken together, the self-fueled nano-penetrator exhibited good biocompatibility and biosafety.

In summary, we elaborately designed and constructed a molecularly self-fueled nano-penetrator for closed-loop treatment of thrombosis and thrombotic disorders. T-BD NAs demonstrated many advantages over conventional gas-driven nanomotors in terms of fabrication simplicity and feasibility, fuel loading capacity, NO generation efficiency, and autonomous and controllable motion feature. More importantly, the in vitro and in vivo results had proven the great potential of T-BD NAs in closed-loop nonpharmaceutical treatment of multiple diseases, including self-navigated, self‐fueled, and self-propelled photothermal/mechanical thrombolysis and NO-mediated thrombus recurrence prevention and ischemic stroke salvage. Such a uniquely fuel pair-engineered nano-penetrator drove a conceptual step forward in modular nanomotor design and provided a facile, safe, and effective nonpharmaceutical modality toward the clinical treatment of thrombotic diseases.

## Methods

### Materials

DiR, coumarin-6 (C6), and thrombin from bovine plasma (37KD) were obtained from Meilun Biotech Co. Ltd., (Dalian, China). BPA, and Sodium nitrite (NaNO_2_) were purchased from Macklin Biochemical Co., Ltd (Shanghai, China). Griess Reagent was obtained from was purchased from Beijing Solarbio Science & Technology Co., ltd. 1, 2-distearoyl-sn-glycero-3-phosphoethanolamine-N-[methoxy(polyethyleneglycol)-2000] (DSPE-PEG_2K_) was purchased from A.V.T. Pharmaceutical Co., Ltd (Shanghai, China). DSPE-PEG_2K_-CREKA was synthesized by Premierbiochem Co., Ltd (Suzhou, China). sCD40L Assay Kit was purchased from Beijing Solarbio Science & Technology Co., ltd. Cell culture dishes and plates were bought from NEST Biotechnology Co., Ltd (Wuxi, China). All solvents and reagents used were analytical or HPLC standard.

### Synthesis of compound BNN6

The synthesize route of N, N’-Di-sec-butyl-N, N’-dinitroso1,4-phenylenediamine (BNN6) was as follows: First, 2.34 mL (10 mmoL) of BPA was added to 18 mL of ethanol, stirring for 30 min under nitrogen protection. Then, 20 mL (6 M) degassed NaNO_2_ aqueous solution was added into the reaction system under nitrogen protection^27^. After stirring 30 min, 20 mL (6 M) HCl aqueous solution was added dropwise through a constant pressure dropping funnel^27^. The reaction solution gradually changed from red to orange and finally obtained a pale-yellow precipitate. After stirring for 4 h, the solid precipitate was collected by centrifugation (1800 × *g*, 10 min)^27^. The collected solid product was washed by pure water and mixed solution of 50% (v/v) ethanol and water in turn 10 times to remove residual reactants, and dried under vacuum drying overnight^27^. All operations are in a dark environment^27^. The structure of synthesized BNN6 was confirmed by a fourier transform ion cyclotron resonance mass spectrometer (FT-MS, solariX, Bruker, Germany) and ^1^H NMR spectroscopy and ^1^C NMR spectroscopy (600 MHz, Bruker AV-600, Germany). solariX was utilized to determine the molecular weight of synthetic BNN6. The type of mass analyzer is the Fourier Transform Ion Cyclotron Resonance (FT-ICR). MS acquisition settings adopted Acquisition Mode: Single MS; Polarity: Positive; Source Accumulation: 0.000 sec; Ion Accumulation Time: 0.280 sec; Laser Power: 20.0 lp; Laser Shot Frequency: 0.001 sec; and Apodization: Full-Sine. High-resolution mass spectrums were acquired by software ftmsControl 2.1 and analyzed by Bruker Compass DataAnalysis 4.4. The validation results were credible when the error between the measured and theoretical molecular weights was not more than 5 ppm. The NMR data were analyzed by MestReC 4.9.9.9 software. ^1^H NMR (400 MHz, CDCl_3_) δ7.50 (4H), 5.05-4.89 (2H), 2.00–1.84 (2H), 1.71-1.49 (2H), 1.25 (t, J = 7.6 Hz, 6H), 1.08 (td, J = 7.4, 5.3 Hz, 6H).^13^C NMR (400 MHz, CDCl_3_) δ128.03, 124.95, 52.05, 28.31, 17.40, 10.07. HRMS (ESI) (m/z) [M + Na] +: calcd. for C_14_H_22_N_4_O_2_: 301.1642, found: 301.1635. The purity was determined by a reverse-phase HPLC system.

### Screening the optimal synergistic dose ratio of BNN6 and DiR

To screen the optimal synergistic dose radio of BNN6 and DiR, we first constructed the bared BNN6 and DiR co-assembly (B-BD NAs) using a nano-precipitation method^47^. In brief, 10 mg of BNN6 and 10 mg of DiR were dissolved in methanol (1 mL), respectively. Then, series mixtures (200 μL) of the above BNN6 Sol and DiR Sol with a wide range of molar ratios (10:1, 8:1, 5:1, 3:1, 2:1, 1:1, 1:2, 1:3, 1:5, 1:8, and 1:10) were added dropwise to purified water (2 mL) under intense stirring (1200 rpm, 3 min) to obtain B-BD NAs. The optimal combination formulation of B-BD NAs was screened out by evaluating NO generation efficiency and particle size. The Griess assay was applied to measure the concentration of generated NO in different molar ratios formulations under 808 nm laser irradiation (2 W/cm^2^, 15 min). The hydrodynamic diameters of B-BD NAs were characterized by Zetasizer (Nano ZS, Malvern Co., UK).

### Preparation and characterization of nanoassemblies

The optimal B-BD NAs (BNN6/DiR = 3:1) was prepared by the nano-precipitation method. Moreover, a mixed solution of DSPE-PEG_2k_-CREKA (10 wt%) and DSPE-PEG_2k_ (15 wt%) was dropwise added into B-BD NAs under intense stirring (1200 rpm, 2 min) to obtain PEGylated targeting modification T-BD NAs. Moreover, N-BD NAs were obtained by the same method, except that BNN6 was replaced with BPA. Finally, the methanol solvent was removed in a vacuum condition at 30 °C. The hydrodynamic diameters and zeta potentials of B-BD NAs, T-BD NAs, and N-BD NAs were characterized by Zetasizer (Nano ZS, Malvern Co., UK). The morphologies of B-BD NAs, T-BD NAs, and N-BD NAs were explored using transmission electron microscopy (TEM, HITACHI, HT7700, Japan) after 2% (w/v) phosphotungstic acid staining.

### Binary co-assembly simulation

The molecular docking simulation method was utilized to investigate the intermolecular interactions between BNN6 and DiR. The 3-dimensional structures of BNN6 and DiR with optimal combination formulation (3:1) were obtained using the Autodock Vina software. The runtime parameters and environment were consistent with the literature^47^. Moreover, the intermolecular interaction breakers (SDS, NaCl, and urea, 50 nM) were co-incubated with the B-BD NAs and T-BD NAs to determine the intermolecular forces. The hydrodynamic diameters of B-BD NAs and T-BD NAs before and after incubation were measured by Zetasizer (NanoZS, Malvern Co., UK).

### Colloidal stability

To detect the colloidal stability of NAs, B-BD NAs, T-BD NAs, and N-BD NAs (equal to 50 µg/mL of DiR) were incubated in PBS (pH 7.4) in a shaking box for 8 h at 37 °C, respectively (*n* = 3). The particle sizes of the above NAs were measured at 0, 2, 4, 6, and 8 h by a Zetasizer (Nano ZS, Malvern Co., UK).

### Long-term stability

To investigate the long-term stability of NAs, B-BD NAs, T-BD NAs, and N-BD NAs (equal to DiR 0.548 mg/mL) were stored at 4 °C under dark conditions^48^. The particle sizes of the above NAs were measured at 1, 7, 18, and 30 days by a Zetasizer (Nano ZS, Malvern Co., UK), respectively.

### In vitro thrombus-targeting capacity

To investigate the thrombus-targeting capacity of T-BD NAs, two artificial thrombus models (platelet-rich plasma clots and whole blood clots) were established, respectively. First, platelet-rich plasma clots were prepared by the following method. Platelet-rich plasma was acquired from SD rats. Then, CaCl_2_ (0.3 M) and thrombin (0.1 U/μL) were utilized to co-incubate with the platelet-rich plasma (150 μL) in a 96-well assay plate for 120 min (37 °C) until thrombosis. Furthermore, whole blood clots were obtained by the following method. The blood (150 μL) was acquired from SD rats and then incubated with CaCl_2_ (0.3 M) as well as thrombin (0.1 U/μL) in a 96-well assay plate for 120 min (37 °C) until thrombosis. The constructed platelet-rich plasma clots and whole blood clots were incubated with PBS, DiR Sol, B-BD NAs, T-BD NAs, and N-BD NAs (50 μL) for 10 min, respectively. Finally, the platelet-rich plasma clots and whole blood clots were washed with PBS (pH 7.4) twice and the fluorescent signals were detected by the in vivo imaging system (IVIS Lumina Series III) (*n* = 3).

### Ultraviolet and fluorescence spectra

The ultraviolet spectra of BNN6 Sol, DiR Sol, B-BD NAs, and T-BD NAs and fluorescence spectra of DiR Sol, B-BD NAs, and T-BD NAs with an equivalent DiR concentration of 20 μg/mL and BNN6 concentration of 16.5 μg/mL were measured by a Varioskan multimode microplate reader (Thermo Scientific, USA).

### In vitro photothermal curves and photothermal conversion efficiency

To investigate the in vitro photothermal curves, PBS, DiR Sol, BNN6 Sol, BD Sol, B-BD NAs, N-BD NAs, and T-BD NAs (DiR 0.548 mg/mL, BNN6 0.452 mg/mL) were exposed to 808 nm laser irradiation (2.0 W/cm^2^) for 15 min. The photothermal curves were obtained by an infrared thermal imaging camera (Fotric 226) (*n* = 3).

To evaluate the photothermal conversion efficiency, T-BD NAs (DiR 0.548 mg/mL, BNN6 0.452 mg/mL) were exposed to laser irradiation (808 nm, 2.0 W/cm^2^) for 10 min and then naturally cooled for 10 min. The temperature variation of T-BD NAs was determined using an infrared thermal imaging camera (Fotric 226). The photothermal conversion efficiency was calculated according to the following literature.

1 $$\eta=\frac{h{{{{{\rm{S}}}}}}\left({T}_{{{{{{\rm{max }}}}}}}-{T}_{{{{{{\rm{surr}}}}}}}\right)-{Q}_{{dis}}}{I\left(1-{10}^{-{A}_{808\,{{{{{\rm{nm}}}}}}}}\right)}$$

The PCE (*η*) was calculated by utilizing the Eq. (1). Where *h* is the heat transfer coefficient of T-BD NAs; S represents the irradiated area; *I* is the laser power density (2.0 W/cm^2^); ${A}_{808{{{{{\rm{nm}}}}}}}$ is the absorbance of T-BD NAs at 808 nm; ${T}_{{{\max }}}$ and ${T}_{{{{{{\rm{surr}}}}}}}$ represents the maximum temperature and the room temperature; ${Q}_{{{{{{\rm{dis}}}}}}}$ is the heat dissipation of the solvent. In Eq. (1), the value of *h*S can be calculated using Eq. (2).

2 $${hS}=\frac{m{C}_{{{{{{\rm{p}}}}}}}}{{\tau }_{s}}$$

The m and *C*p are the mass and the heat capacity (pure water, 4.2 J/g), respectively, and *τ*_*s*_ is the system time constant, calculated by the following Eq. (3).

3 $$t=-{\tau }_{s}\,{{{{{\rm{ln}}}}}}\,\theta$$

The *t* and *θ* represent the cooling time and the dimensionless driving force temperature, respectively. By calculating *t* to the negative natural logarithm of temperature ($-{{{{{\rm{ln}}}}}}\,\theta$), which was linear fitting calculated by the following Eq. (4).

4 $$\theta=\frac{T-{T}_{{{{{{\rm{surr}}}}}}}}{{T}_{\max }-{T}_{{surr}}}$$

T is the surrounding temperature. The *τ*_s_ was calculated to be 166.83.

5 $${Q}_{{dis}}={h}_{0}{{{{{\rm{S}}}}}}\left({T}_{{{\max }},{{{{{\rm{water}}}}}}}-{T}_{{{{{{\rm{surr}}}}}},{{{{{\rm{water}}}}}}}\right)$$

${Q}_{{{{{{\rm{dis}}}}}}}$ was calculated by the Eq. (5). According to the obtained data, the photothermal conversion efficiency of T-BD NAs was calculated to be 42.7%.

### NO generation capacity assessment

In order to detect the NO generation efficiency, the NO concentration was quantitatively measured according to NO kit (Griess assay). Typically, DiR Sol, BD Sol, B-BD NAs, T-BD NAs, and N-BD NAs (0.548 mg/mL of DiR and 0.452 mg/mL of BNN6, 800 μL) were added into a 1.5 mL brown EP tubes and irradiated the liquid level directly from above the brown EP tubes under 808 nm laser (2 W/cm^2^) at more than one-time points (5, 10, 20, 30, 60 min). The samples (100 μL) were removed and seeded into 96-well plates for Griess assay. The principle of Griess assay was to measure the concentration of NaNO_2_ and the calibration curve has been presented in Supplementary Fig. 6. Sample preparations and tests strictly followed the manufacturer’s protocol (*n* = 3).

### Autonomous motion features assessment

The BNN6 and DiR co-loaded PLGA NPs (PLGA-BD NPs) were prepared using the emulsion solvent evaporation technique. PLGA (60 mg), DiR (2.35 mg), and BNN6 (2.19 mg) were dissolved in 4 mL mixed solvent (dichloromethane/tetrahydrofuran = 3:1, v/v). The mixed solvent (2 mL) was added into an aqueous solution (4 mL) containing 1% PVA and followed by sonication for 6 min at 380 W in the ice bath. Then, the organic solvent in the emulsion was removed by vacuum-rotary evaporation for 3 h at 37 °C. The nanoparticle suspensions were centrifuged for 30 min at 15000 × *g* and washed thrice and finally dispersed into purified water.

The Positive Fluorescence Microscope with Nikon camera (×40) was employed to record the movement trajectories of the nano-penetrator. To detect the motion of NAs, a bright-field condenser was kept open to provide visible light for imaging. The 808 nm light was utilized to achieve the motion of the T-BD NAs. Typically, T-BD NAs and PLGA-BD NPs (the total concentration of DiR and BNN6 was 1.0 mg/mL) were irradiated in 808 nm laser under short-time laser irradiation at different laser densities (0.5 W/cm^2^–2 W/cm^2^, 60 s). Moreover, to further evaluate the motion behavior of nano-penetrator in a physiological environment, T-BD NAs (equal to 50 µg/mL of DiR) were incubated with PBS (pH 7.4) or PBS (pH 7.4) containing 10% FBS with short-time laser irradiation (808 nm, 2 W/cm^2^, 60 s)^49^. The motion movies were analyzed by software Image J to obtain the movement trajectories and speed of the nano-penetrator (*n* = 3).

### In vitro nonpharmaceutical thrombolysis

The whole blood clots were prepared to evaluate the nonpharmaceutical thrombolysis effect. First, blood (50 μL) harvested from SD rats, thrombin (5 U/mL), and CaCl_2_ (3 mM) were mixed into 200 μL EP tubes and incubated for 4 h at 37 °C^13^ to induce thrombosis. The prepared blood clots were placed into a 5 mL glass vial and the added 4.0 mL PBS as well as 1.0 mL formulations (DiR Sol, BNN6 Sol, BD Sol, B-BD NAs, T-BD NAs, and N-BD NAs (0.548 mg/mL of DiR and 0.452 mg/mL of BNN6)). Then the blood clots were irradiated by 808 nm laser for 20 min (2 W/cm^2^). After irradiation, the thrombolysis effect could be observed in the glass vials. Then the supernatant was collected and the absorbance of fibrin OD_415_ and hemoglobin OD_540_ were measured by Varioskan LUX multimode microplate reader (Thermo Scientific, USA). Moreover, the blood clots before and after thrombolytic treatment were dried and weighed to access the thrombolytic rate (*n* = 3). (Thrombolytic rate = (thrombus weight before therapy−thrombus weight after therapy)/thrombus weight before therapy).

### Photothermal/mechanical synergism thrombus deep penetration

A small blot thrombus model was obtained to evaluate the penetration and retention features of the fuel pair NAs. Fresh blood obtained from SD rats (15 μL) was seeded into the bottom of EP tubes (200 μL) and incubated 3 h at 37 °C to induce thrombosis. Moreover, to track the NAs in thrombus the C6 was utilized to label the NAs and obtained C6/B-BD NAs, C6/N-BD NAs, and C6/T-BD NAs, and C6 Sol was utilized as a control. To prepare C6/T-BD NAs, the mixed methanol Sol of DiR (109.6 μL,10 mg/mL), BNN6 (90.4 μL,10 mg/mL), and C-6 (50 μL, 1 mg/mL) were dripped into purified water (2 mL) under intense stirring (1200 rpm, 3 min) and subsequent processes were same as the preparation method of T-BD NAs. Moreover, the preparation process of C6/B-BD NAs was similar to C6/T-BD except for without adding DSPE-PEG_2K_ and DSPE-PEG_2K_-CREKA. C6/N-BD NAs were prepared by the same method, except that BNN6 was replaced with BPA. The prepared blood clot was incubated with the above formulations for 30 min and irradiated with the 808 nm laser for 15 min (2 W/cm^2^), respectively. Finally, the clots were washed thrice with PBS (pH 7.4) and the fluorescent signals in the thrombus interior were measured by the confocal laser scanning microscope (*n* = 3) (Confocal laser scanning microscopy: NIS 4.13, Nikon, Japan). And then, the clots were collected and examined by Fluorescence imaging to further evaluate the thrombus penetration effect.

In addition, a larger whole blood clot was also conducted as described in “In vitro nonpharmaceutical thrombolysis”, to further evaluate the thrombus penetration ability of the fuel pair NAs. Then different formulations (DiR Sol, BNN6 Sol, BD Sol, B-BD NAs, T-BD NAs, and N-BD NAs (0.548 mg/mL of DiR and 0.452 mg/mL of BNN6) were co-incubated with the blood clots under the 808 nm laser irradiation (2 W/cm^2^, 20 min). The treated blood clots were collected and examined by H&E staining.

### Cell lines and animal studies

HUVEC was obtained from Cyagen Biosciences. Biotechnology Development Co., Ltd HUVEC lines validation using short tandem repeat (STR) markers were performed by Shanghai Qingqi Biotechnology Development Co., Ltd. Sample DNA was extracted using Axygen’s genome extraction kit and amplified using a 10-STR amplification protocol (D4S2408 as the human locus). STR loci were detected on an ABI model 3730XL genetic analyzer. Data were analyzed using Gene Mapper ID 3.2 software (Applied Biosystems). Appropriate positive and negative controls were run and confirmed for each sample submitted.

SD rats (male, 6 weeks old) and Kunming (KM) mice (female, 11 weeks old) were utilized and conformed to the Animal Laboratory Ethics Committee of Shenyang Pharmaceutical University. The living environment of animals was maintained at a temperature of ~25 °C with a 12 h light/dark cycle, with free access to standard food and water. All the animal experiments were conducted according to the Guidelines for the Care and Use of Laboratory Animals approved by the Institutional Animal Ethical Care Committee (IAEC) of Shenyang Pharmaceutical University.

### Pharmacokinetics

To explore pharmacokinetics behavior, DiR Sol, B-BD NAs, T-BD NAs, and N-BD NAs (DiR 5 mg/kg) were intravenously injected into SD rats (male, 6 weeks old), respectively (180–220 g) (*n* = 5). At the specified time intervals (0.05, 0.083, 0.25, 0.5, 1, 2, 4, 6, 8, and 12 h), the blood (~500 µL) was collected from rats and centrifuged (11,000 × *g*, 3 min) to obtain plasma. DiR was then extracted from plasma by protein precipitation method. Finally, the fluorescent intensity of DiR in plasma samples was measured using the multimode microreader (Thermo Scientific, USA) DAS 2.1.1 software was used to calculate the pharmacokinetic parameters.

### Arteries thrombosis vessels-targeting fluorescence imaging

The FeCl_3_-induced rat carotid arterial thrombosis model was used for arterial thrombosis vessels-targeting fluorescence imaging. First, all the SD rats (male, 6 weeks old, 180–220 g) were anesthetized by intraperitoneal injection of 3% pentobarbital (30 mg/kg) and fixed on an operating table. A midline incision was made between the chin and sternum, and the peripheral muscles were separated to expose the common carotid artery (CCA). Then, a 10% FeCl_3_-soaked filter paper (10 × 10 mm) was wrapped on the exposed CCA for 8 min to induce thrombosis. Finally, the embolization of the CCA was washed using PBS to remove the residual FeCl_3_. Then, the rats were intravenously injected with DiR Sol, B-BD NAs, T-BD NAs, and N-BD NAs at a DiR equivalent dose of 5 mg/kg, respectively (*n* = 5). At specified time intervals (5, 15, 30, 60, 90, and 120 min), the embolic vessels were imaged using a noninvasive optical imaging system (IVIS Lumina Series III).

### In vivo photothermal efficacy in arteries thrombosis vessels

The FeCl_3_-induced arterial thrombosis rats were constructed to investigate the in vivo photothermal efficacy. The model rats were intravenously administered with PBS, DiR Sol, B-BD NAs, T-BD NAs, and N-BD NAs at a DiR equivalent dose of 5 mg/kg, respectively. After administration, the thrombus sites of model rats were exposed to 808 nm NIR laser at optimal drug accumulation time point (DiR Sol group at 5 min, B-BD NAs group at 30 min, N-BD NAs and T-BD NAs group at 2 h) (*n* = 5). The light treatment lasted for 15 min. The infrared thermal imaging camera (Fotric 226) was used to record the thermal images and local temperature variations of the above groups.

### In vivo nonpharmaceutical thrombolysis in FeCl_3_-induced arterial thrombosis model

The SD rats (male, 6 weeks old) were randomly divided into eight groups (*n* = 5): (i) Sham group; (ii) Embolism group; (iii) DiR Sol group; (iv) BD Sol group; (v) B-BD NAs group; (vi) N-BD NAs group; (vii) T-BD NAs group and (viii) LBK group. As previously mentioned, the embolic models were established and injected with different formulations (DiR equivalent dose of 5 mg/kg and BNN6 of 4.125 mg/kg). According to the previous experiment results, DiR sol group was exposed to 808 nm NIR laser for 5 min, B-BD NAs group at 30 min, and N-BD NAs and T-BD NAs group at 2 h. The thrombus site of the rats was subjected to light treatment (808 nm, 2.0 W/cm^2^) for 15 min. After 1 h, the above group rats were sacrificed, and the carotid vessels were collected and examined by H&E staining to evaluate the thrombus treatment effect. The embolism area of images was quantified using ImageJ 1.8.0 software and analysis by GraphPad prism 8.0 software.

Moreover, FeCl_3_-induced carotid arterial thrombosis rats were used to evaluate whether the thrombolytic fragments produced after T-BD NAs treatment could induce small vessel blockage. After thrombosis, T-BD NAs were intravenously injected into rats at a DiR equivalent dose of 5 mg/kg. The treatment process was consistent with previous experiment methods. After 14 days, the primary organ (heart, liver, spleen, lung, kidney) of rat were separated to stain by H&E to evaluate the small vessel blockage and tissue damage. Then, the H&E staining sections of carotid artery embolization in rats with or without T-BD NAs treatment under laser irradiation were obtained.

### In vivo antiplatelet activity in FeCl_3_-induced arterial thrombosis model

Blood samples acquired from SD rats (male, 6 weeks old) after the above-mentioned treatments were collected into a tube and centrifuged (11,000 × *g*, 10 min) to obtain plasma. The level of sCD40L was measured with a mouse sCD40L ELISA kit (Mlbio, Shanghai, China), according to manufacturer’s instructions (*n* = 5).

### Targeting fluorescence imaging in mouse tail thrombosis model

Kunming (KM) mice (female, 11 weeks old) were starved for 12 h and intraperitoneally injected with fresh carrageenan (20 mg/kg) to induce tail venous thrombolysis at 20 °C. One day later, the tail end of the mice turned black, suggesting successful thrombosis. Then, the mice were intravenously injected with DiR Sol, B-BD NAs, T-BD NAs, and N-BD NAs at a DiR equivalent dose of 5 mg/kg and 4.125 mg/kg of BNN6, respectively. At specified time intervals (5, 15, 30, 60, 90, 120, 180, and 240 min), the fluorescence signals of the tails thrombus were observed using a noninvasive optical imaging system (*n* = 5) (IVIS Lumina Series III).

### In vivo photothermal efficacy in mouse tail thrombosis model

Furthermore, the photothermal efficacy in the tail thrombosis model was constructed to evaluate the in vivo photothermal efficacy of NAs. The mouse tail thrombosis model was established. T-BD NAs (DiR 5 mg/kg) and PBS were intravenously administered to the model mice. The 808 nm laser irradiation was imposed on the black tail at 3 h after administration. The infrared thermal imaging camera (Fotric 226) was used to record the thermal images and local temperature variations of the above groups.

### In vivo nonpharmaceutical thrombolysis in mouse tail thrombosis model

The mouse tail thrombosis model was established. The KM mice (female, 11 weeks old) were divided randomly into seven groups and intravenously injected with 100 μL of PBS, DiR Sol, B-BD NAs, T-BD NAs, N-BD NAs (DiR equivalent dose of 5 mg/kg, and 4.125 mg/kg of BNN6), and LBK (8000 U/kg) via tail veins every other day for a total of four injections, respectively (*n* = 5). After administration, according to the mouse tail thrombosis fluorescence imaging experiment, the DiR sol group was exposed to 808 nm NIR laser at 5 min, B-BD NAs group at 15 min, and N-BD NAs and T-BD NAs group at 3 h. The light treatment lasted for 15 min. The tail length of the tails was measured daily. After 9 days, the mice were sacrificed and collected plasma to measure the coagulation indicators of AT, APTT, and FIB. The tails were clipped off and collected into 4% paraformaldehyde and examined by H&E to evaluate the thrombus treatment effect.

### In vivo prevention of recurrent thrombosis

Nine days after the end of treatment, the healed mice treated with T-BD NAs or LBK were intraperitoneally injected with fresh carrageenan (20 mg/kg) and fed at 20 °C. The length of the black tails was recorded every day. After 3 days, all mice were sacrificed and the tails were clipped off and collected into 4% paraformaldehyde for H&E staining (*n* = 5).

### Ischemic stroke salvage

The rat model of middle cerebral artery occlusion/reperfusion (MCAO) was established to detect the in vivo ischemic stroke salvage of T-BD NAs. Briefly, all the SD rats (male, 6 weeks old, 220–260 g) were anesthetized by intraperitoneal injection 3% pentobarbital (30 mg/kg) and fixed on the heating blanket to maintain the surrounding temperature at approximately 37 °C during surgery. The CCA, internal carotid artery (ICA) and external carotid artery (ECA) were exposed and isolated. The proximal end of the CCA was blocked via a suture, and the filament with silicone was inserted from the ECA to the ICA for 90 min. The reperfusion of the ischemic area was achieved by suture removal.

To investigate the brain-specific fluorescence imaging, the head hair of healthy SD rats and MCAO SD rats were shaved. Then, DiR Sol and T-BD NAs at a DiR equivalent dose of 5 mg/kg were intravenously injected into MCAO SD rats and equivalent T-BD NAs were intravenously injected into healthy SD rats (*n* = 5). At specified time intervals (5, 15, 30, 60, 90, 120, and 180 min), the fluorescence signal of heads was detected using a noninvasive optical imaging system (IVIS Lumina Series III). For quantitative analysis of the fluorescence intensity of major organs, the rats were sacrificed at 2 h after administration, and heart, liver, spleen, lung, kidney, and brain were acquired to detect the fluorescence signal by the IVIS Lumina Series III.

For the neuro-score evaluation and infarct area measurement, the SD rats were randomly divided into four groups (*n* = 5): (i) Sham group; (ii) Embolism group; (iii) DiR Sol group; (iv) T-BD NAs group. Groups (iii) and (iv) group were injected with DiR Sol and T-BD NAs (DiR equivalent dose of 5 mg/kg). After administration, 808 nm laser irradiation was applied to groups (iii) and (iv) at 15 min and 2 h, respectively. After 24 h, the universal five-point-scale method was utilized to measure the neuroscore. Then, the rats were euthanized and the brain tissues were collected and washed with PBS (pH 7.4) three times, and immediately frozen at −20 °C for 15 min. Finally, the brain tissues were cut into four 2 mm thick sections and co-incubated with PBS containing 2% 2,3,5-triphenyl tetrazolium chloride (TTC) at 37 °C for 20 min. The TTC-stained brain tissues were fixed in 4% paraformaldehyde for photographing. Moreover, the brain tissues were collected and stained by H&E to evaluate the pathological changes. The quantification analysis was conducted by software ImageJ to obtain the infarct area of the coronal section.

### Safety evaluation

For the in vitro hemolysis evaluation, the collected rat RBCs (50 μL) were dispersed in PBS (1 mL) to obtain an erythrocyte suspension. BNN6 Sol, DiR Sol, BD Sol, B-BD NAs, T-BD NAs, and N-BD NAs at the same concentrations (DiR 1 mM and BNN6 3 mM, 50 μL) were incubated with the erythrocyte suspension with or without 808 nm laser irradiation (2 W/cm^2^, 5 min) for 3 h at 37 °C. After centrifugation (1800 × *g*, 5 min), the supernatant was collected and UV absorbance of hemoglobin at 540 nm was analyzed using a microplate reader. (*n* = 3) (PBS: negative control, pure water: positive control).

Moreover, for the in vitro cytotoxicity assays, HUVEC (4 × 10^3^ cells/well) were seeded into 96-well plates and incubated for 12 h. And then different concentrations (0, 25, 50, 100, and 200 μM) of BNN6 Sol, DiR Sol, BD Sol, B-BD NAs, T-BD NAs, or N-BD NAs, were added into the HUVEC. The last five groups were all set to light and non-light groups. After incubation for 4 h, the light groups were irradiated with a laser (808 nm, 2 W/cm^2^) for 5 min. All groups were cultured for a further 44 h in dark conditions. Thereafter, 25 µL of MTT (5 mg/mL in PBS) was added to the culture medium and further incubated for 4 h at 37 ° C. Finally, the supernatant was discarded and replaced with 200 µL of dimethyl sulfoxide, and the absorbency at 490 nm was measured using Varioskan LUX multimode microplate reader (*n* = 3) (Thermo Scientific, USA).

Furthermore, in vivo biosafety of T-BD NAs was also evaluated. The DiR sol, BD Sol, B-BD NAs, T-BD NAs, N-BD NAs (DiR equivalent dose of 5 mg/kg), and LBK (8000 U/kg) were intravenously injected into the healthy SD rats, respectively. After administration, according to the arterial thrombosis vessels-targeting fluorescence imaging experiment, DiR sol group was exposed to 808 nm NIR laser at 5 min, B-BD NAs group at 30 min, and N-BD NAs and T-BD NAs group at 2 h (*n* = 5). The light treatment was continued for 15 min. One day later, the rats were sacrificed, and the plasma and main tissues were harvested. The plasma levels of ALT, AST, BUN, and creatinine were measured by the corresponding ELISA (enzyme-linked immunosorbent assay) kits (Nanjing Jiancheng). Moreover, the major organ tissues (heart, liver, spleen, lung, and kidney) were collected to stain by H&E to evaluate the pathological changes. Moreover, FeCl_3_-induced rat carotid arterial thrombosis models were used to examine the potential blockage lesions caused by the thrombolytic fragments produced after T-BD NAs treatment. After thrombosis, T-BD NAs were intravenously injected into rats at a DiR equivalent dose of 5 mg/kg. The treatment process was consistent with previous experimental methods. After 14 days, the primary organs (heart, liver, spleen, lung, and kidney) of rats were collected and stained with H&E to evaluate small vessel blockage and tissue damages.

### Statistical analysis

All data were calculated using GraphPad Prism and expressed as mean ± standard deviation (SD). The significant differences between groups were analyzed by *T* test or one-way analysis of variance (ANOVA). The exact *P* value is provided in the corresponding figure, and p values less than 0.05 was considered statistically significant.

### Reporting summary

Further information on research design is available in the Nature Portfolio Reporting Summary linked to this article.

## Supplementary information

Supplementary Information

Description of Additional Supplementary Files

Supplementary Movie 1

Supplementary Movie 2

Reporting Summary

## Data Availability

All data supporting the findings of this study are available within the Article, Supplementary Information, or Source Data file. The source data underlying Fig. 2c, d, g, h, j–l, Fig. 3c, d, f, g, i–k, Fig. 4c, f, h, Fig. 5c, d, f, i, Fig. 6c, d, h, Fig. 7c, d, f, h, Supplementary Fig. 4, 5b, 6, 7, 8, 9, 10, 12, 13b, 15, 16, 20, 23, 24, and 25 have been deposited in the Figshare database (10.6084/m9.figshare.21801139)^50^. Source Data.xlsx (figshare.com) Source data are provided with this paper.

## Source data

Source Data

## References

[1] Mackman N (2008). Triggers, targets and treatments for thrombosis. Nature.

[2] Heit JA (2015). Epidemiology of venous thromboembolism. Nat. Rev. Cardiol..

[3] Khan F, Tritschler T, Kahn SR, Rodger MA (2021). Venous thromboembolism. Lancet.

[4] Boeckh-Behrens T (2016). Thrombus histology suggests cardioembolic cause in cryptogenic stroke. Stroke.

[5] Wendelboe AM, Raskob GE (2016). Global burden of thrombosis: epidemiologic aspects. Circ. Res..

[6] Tao T (2020). Natural medicine in neuroprotection for ischemic stroke: challenges and prospective. Pharmacol. Ther..

[7] May JE, Moll S (2020). How I treat unexplained arterial thrombosis. Blood.

[8] Su M (2020). Nano-medicine for thrombosis: a precise diagnosis and treatment strategy. Nano-Micro Lett..

[9] Zhao Z (2022). Elaborately engineering a self-indicating dual-drug nanoassembly for site-specific photothermal-potentiated thrombus penetration and thrombolysis. Adv. Sci..

[10] Liu J (2021). Dendrimeric nanosystem consistently circumvents heterogeneous drug response and resistance in pancreatic cancer. Exploration.

[11] Myerson JW (2016). Non-affinity factors modulating vascular targeting of nano- and microcarriers. Adv. Drug Deliv. Rev..

[12] Zhao Z (2020). Emerging nanotherapeutics for antithrombotic treatment. Biomaterials.

[13] Liu, R. et al. Advances of nanoparticles as drug delivery systems for disease diagnosis and treatment. *Chin. Chem. Lett.* **34**, 107518 (2022).

[14] Frohlich E (2017). Hemocompatibility of inhaled environmental nanoparticles: potential use of in vitro testing. J. Hazard. Mater..

[15] Deng Q (2021). Biological mediator-propelled nanosweeper for nonpharmaceutical thrombus therapy. ACS Nano.

[16] Wu Z (2021). Multi-pathway microenvironment regulation for atherosclerosis therapy based on beta-cyclodextrin/L-arginine/Au nanomotors with dual-mode propulsion. Small.

[17] Gao C (2021). Biomedical micro-/nanomotors: from overcoming biological barriers to in vivo imaging. Adv. Mater..

[18] Wan M (2019). Bio-inspired nitric-oxide-driven nanomotor. Nat. Commun..

[19] Carpenter AW, Schoenfisch MH (2012). Nitric oxide release: part II. Therapeutic applications. Chem. Soc. Rev..

[20] Naghavi N, de Mel A, Alavijeh OS, Cousins BG, Seifalian AM (2013). Nitric oxide donors for cardiovascular implant applications. Small.

[21] Tao Y (2022). Nitric oxide-driven nanomotors with bowl-shaped mesoporous silica for targeted thrombolysis. J. Colloid Interface Sci..

[22] Zhang Y (2021). Self‐powered technology based on nanogenerators for biomedical applications. Exploration.

[23] Wu Z (2022). Multi‐pathway microenvironment regulation for atherosclerosis therapy based on beta‐cyclodextrin/l‐arginine/Au nanomotors with dual‐mode propulsion. Small.

[24] Li, T. et al. A universal chemotactic targeted delivery strategy for inflammatory diseases. *Adv. Mater.* **34**, 2206654 (2022). 10.1002/adma.202206654 36122571

[25] Nielsen VG, Baird MS, Chen L, Matalon S (2000). DETANONOate, a nitric oxide donor, decreases amiloride-sensitive alveolar fluid clearance in rabbits. Am. J. Respir. Crit. Care Med..

[26] Yang Z (2020). Fighting immune cold and reprogramming immunosuppressive tumor microenvironment with red blood cell membrane-camouflaged nanobullets. ACS Nano..

[27] Fan J (2016). Light-responsive biodegradable nanomedicine overcomes multidrug resistance via NO-enhanced chemosensitization. ACS Appl. Mater. Interfaces.

[28] An, H. W. et al. Rationally designed modular drug delivery platform based on intracellular peptide self‐assembly. *Exploration* **1**, 20210153 (2021). 10.1002/EXP.20210153 PMC10190849 37323217

[29] Zhang L (2016). Prolonging the plasma circulation of proteins by nano-encapsulation with phosphorylcholine-based polymer. Nano Res..

[30] Li, S. et al. Precisely engineering a carrier-free hybrid nanoassembly for multimodal DNA damage-augmented photodynamic therapy. *Chem. Eng. J.* **426**, 130838 (2021).

[31] Yang, F. et al. Precisely engineering a dual-drug cooperative nanoassembly for proteasome inhibition-potentiated photodynamic therapy. *Chin. Chem. Lett.* **33**, 1927–1932 (2021).

[32] Zhang X (2020). Emerging carrier-free nanosystems based on molecular self-assembly of pure drugs for cancer therapy. Med. Res. Rev..

[33] Kaur S (2022). Methods to detect nitric oxide and reactive nitrogen species in biological sample. Methods Mol. Biol..

[34] Wu Ziyu (2022). Carrier-free trehalose-based nanomotors targeting macrophages in inflammatory plaque for treatment of atherosclerosis. ACS Nano.

[35] Zhang F (2019). Metal-organic-framework-derived carbon nanostructures for site-specific dual-modality photothermal/photodynamic thrombus therapy. Adv. Sci..

[36] Xiao F (2020). Photosensitizer conjugate-functionalized poly(hexamethylene guanidine) for potentiated broad-spectrum bacterial inhibition and enhanced biocompatibility. Chin. Chem. Lett..

[37] Wan M (2020). Platelet-derived porous nanomotor for thrombus therapy. Sci. Adv..

[38] Massberg S (1999). Increased adhesion and aggregation of platelets lacking cyclic guanosine 3′, 5′-monophosphate kinase I. J. Exp. Med..

[39] Zhang F (2019). Metal–organic‐framework‐derived carbon nanostructures for site‐specific dual‐modality photothermal/photodynamic thrombus therapy. Adv. Sci..

[40] Castillo J, Rama R, Dávalos A (2000). Nitric oxide–related brain damage in acute ischemic stroke. Stroke.

[41] Obeidat M (2012). Glomerular endothelium: a porous sieve and formidable barrier. Exp. Cell Res..

[42] Li M (2020). Platelet membrane biomimetic magnetic nanocarriers for targeted delivery and in situ generation of nitric oxide in early ischemic stroke. ACS Nano.

[43] Kassner A, Merali Z (2015). Assessment of blood-brain barrier disruption in stroke. Stroke.

[44] He L (2020). Highly bioactive zeolitic imidazolate framework-8-capped nanotherapeutics for efficient reversal of reperfusion-induced injury in ischemic stroke. Sci. Adv..

[45] Xu J (2019). Sequentially site-specific delivery of thrombolytics and neuroprotectant for enhanced treatment of ischemic stroke. ACS Nano.

[46] Lu Y (2019). Microthrombus-targeting micelles for neurovascular remodeling and enhanced microcirculatory perfusion in acute ischemic stroke. Adv. Mater..

[47] Wang Y (2021). Investigating the crucial roles of aliphatic tails in disulfide bond-linked docetaxel prodrug nanoassemblies. Asian J. Pharm. Sci..

[48] Zhang H (2022). NIR‐II hydrogen‐bonded organic frameworks (HOFs) used for target‐specific amyloid‐β photooxygenation in an alzheimer’s disease model. Angew. Chem. Int. Ed. Engl..

[49] Cui T (2020). Self-propelled active photothermal nanoswimmer for deep-layered elimination of biofilm in vivo. Nano Lett..

[50] Zhang, H. et al. Molecularly self‐fueled nano-penetrator for nonpharmaceutical treatment of thrombosis and ischemic stroke. figshare. Dataset. 10.6084/m9.figshare.21801139 (2023). 10.1038/s41467-023-35895-5 PMC9845202 36650139

