# Supplementary material for: Molecularly self‐fueled nano-penetrator for nonpharmaceutical treatment of thrombosis and ischemic stroke
Source: Nat Commun. 2023 Jan 17;14:255. doi: 10.1038/s41467-023-35895-5 (PMC9845202; doi:10.1038/s41467-023-35895-5)
Supplement: Supplementary file 1 — Supplementary Information [file 41467_2023_35895_MOESM1_ESM.pdf]

Supplementary Information

Title:

Molecularly Self-Fueled Nano-Penetrator for Nonpharmaceutical Treatment of Thrombosis and Ischemic Stroke

Authors: Hongyuan Zhang[1#], Zhiqiang Zhao[1#], Shengnan Sun[1], Sen Zhang[2,] Yuequan Wang[1], Xuanbo Zhang[1], Jin Sun[1], Zhonggui He[1], Shenwu Zhang[1*], Cong Luo[1*]

Affiliations:

[1]Department of Pharmaceutics, Wuya College of Innovation, Shenyang Pharmaceutical University, Shenyang 110016, P. R. China

[2]Key Laboratory of Structure-Based Drug Design and Discovery of Ministry of Education, Shenyang Pharmaceutical University, Shenyang 110016, P. R. China

***Corresponding authors:**

Cong Luo, Ph.D. and Shenwu Zhang, Ph.D.

Department of Pharmaceutics, Wuya College of Innovation, Shenyang Pharmaceutical University, No. 103 Wenhua Road, Shenyang 110016, P. R. China

E-mail: luocong@syphu.edu.cn; zhangshenwu@aliyun.com

[#]These authors contributed equally to this work.

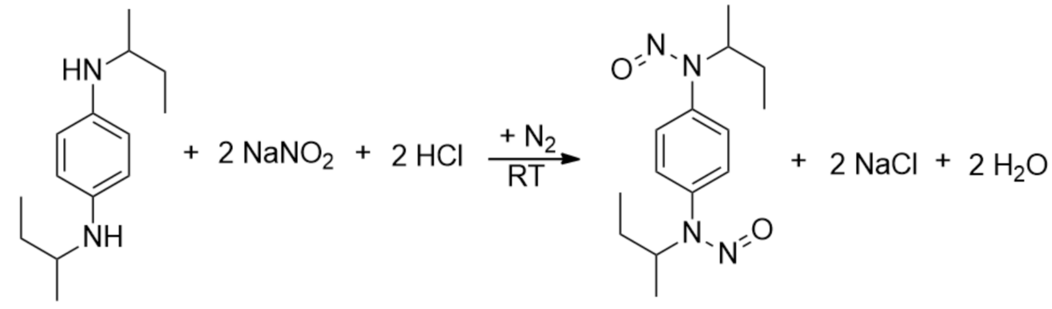

BPA **BNN6**

Supplementary Fig. 1 Synthesis route of BNN6.

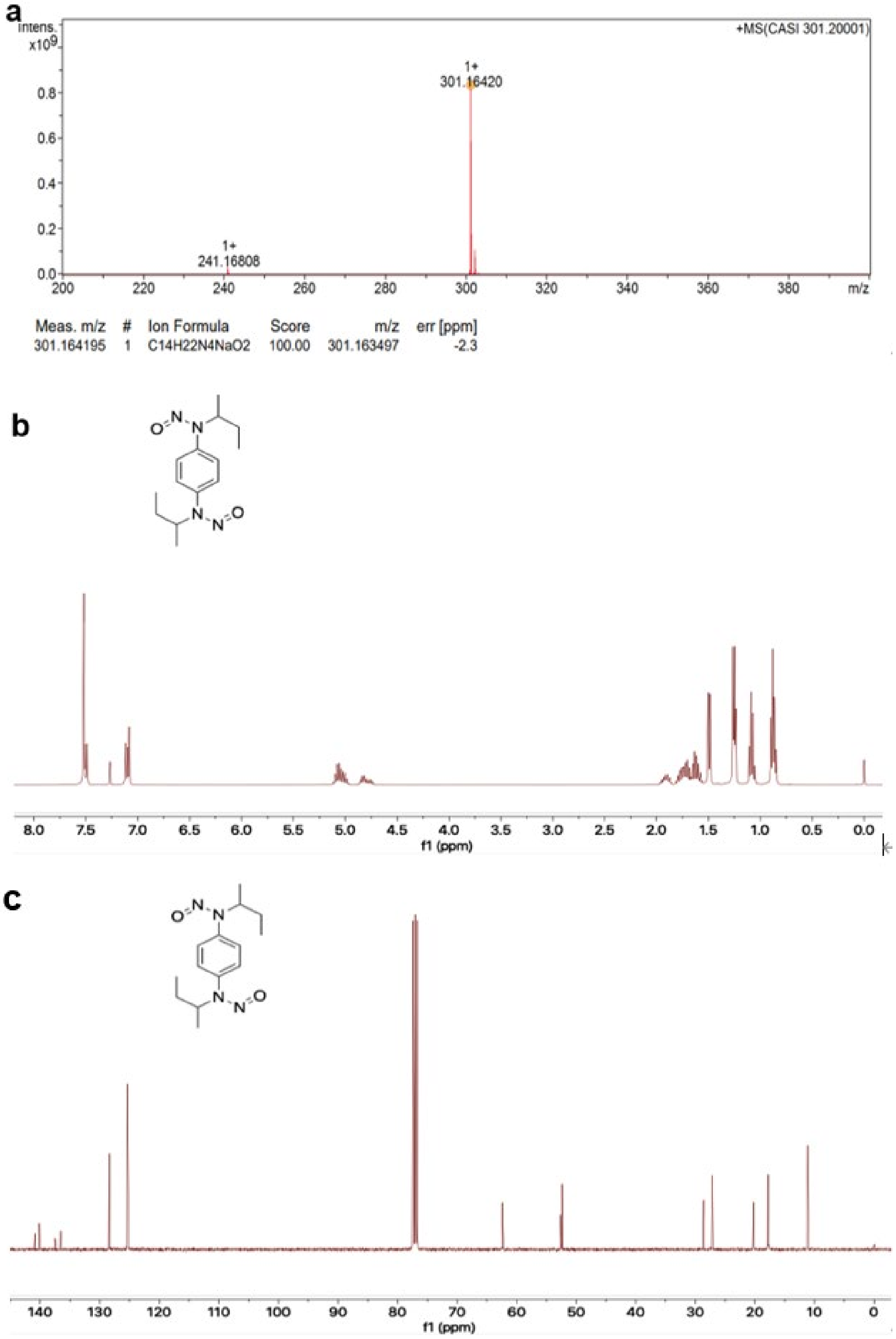

Supplementary Fig. 2 (**a**) Mass spectrometry result of BNN6. (**b**) H NMR spectra of BNN6(400 MHz, CDCl3) δ7.50 (4H), 5.05-4.89 (2H), 2.00-1.84 (2H), 1.71-1.49 (2H), 1.25 (t, J =7.6 Hz, 6H), 1.08 (td, J = 7.4, 5.3 Hz, 6H). (**c**) 13C NMR spectra of BNN6 (400 MHz, CDCl3) δ128.03, 124.95, 52.05, 28.31, 17.40, 10.07.

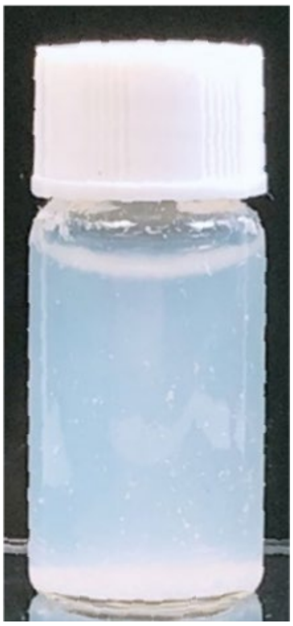

Supplementary Fig. 3 Aqueous dispersion of BNN6.

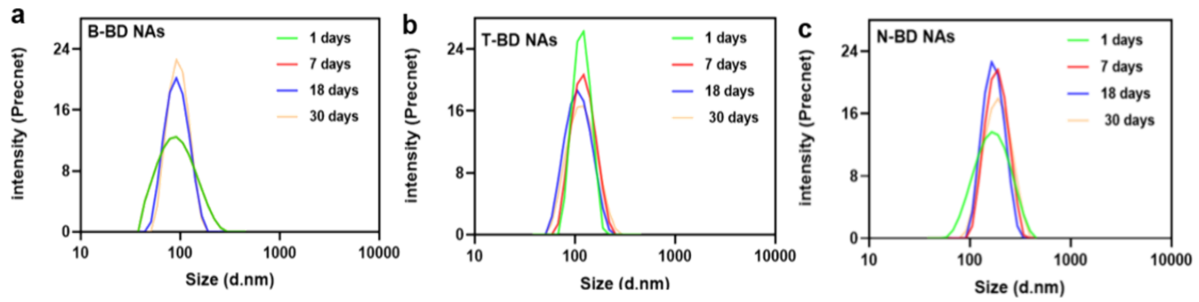

Supplementary Fig. 4 Long-term stability of (**a**) B-BD NAs, (**b**)T-BD NAs, and (**c**)N-BD NAs stored at 4°C under dark conditions.

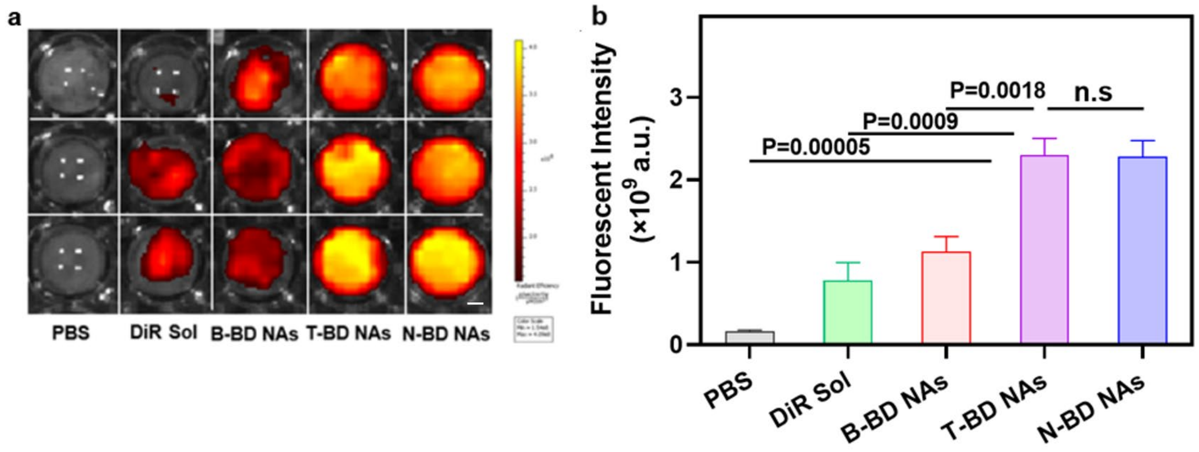

Supplementary Fig. 5 (**a**) Representative In vitro thrombus-targeting fluorescence images (scale bar = 100 μm) and (**b**) quantitative results in artificial whole blood clots treated with different formulations Data are presented as mean ± SD (n=3 independent experiments) Source data are provided as a Source Data file. One-way ANOVA (one-sided) with Dunnett's multiple comparisons test was used for the analysis of data and adjusted P value.

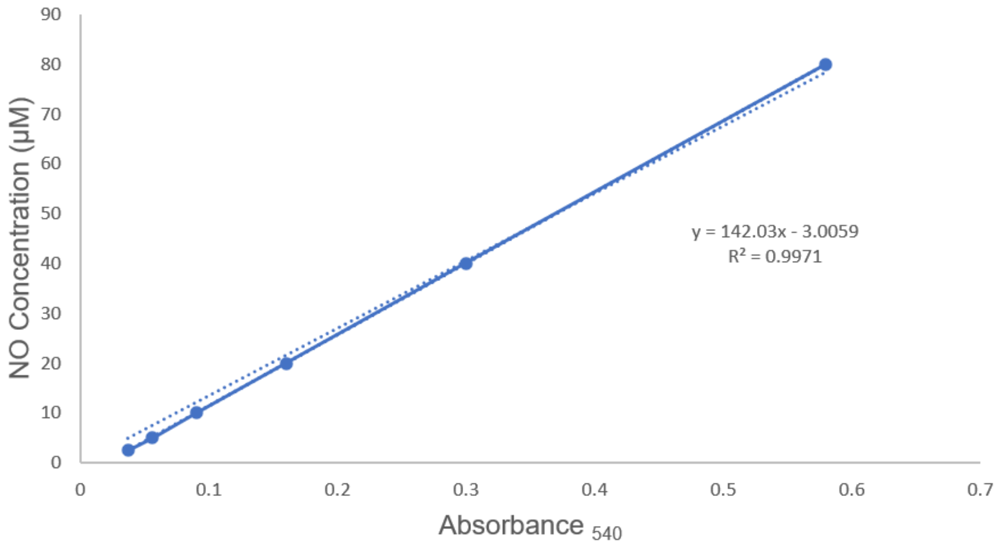

Supplementary Fig. 6 Concentration-absorbance calibration curve of $NaNO_2$ determined by the Griess assay at 540 nm.

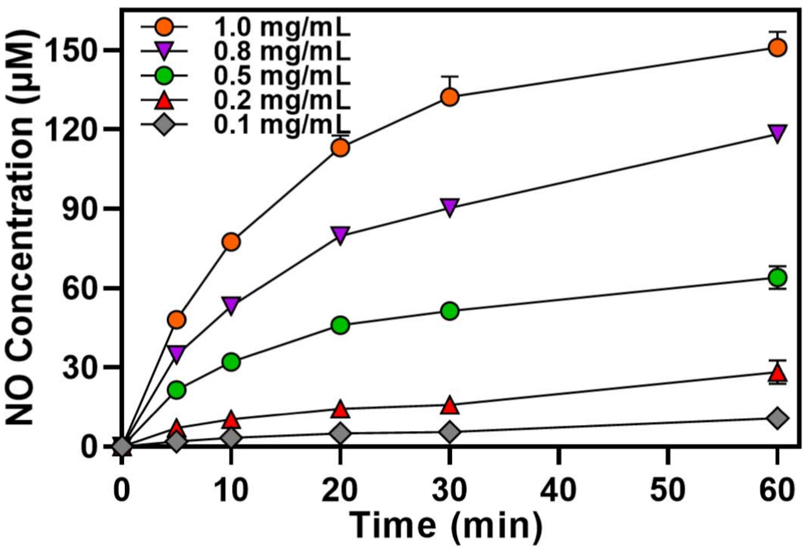

Supplementary Fig. 7 NO generation from T-BD NAs at different concentrations of BNN6 under 808 nm laser irradiation ($2W/cm^2$), Data are presented as mean ± SD (n=3 independent experiments) Source data are provided as a Source Data file.

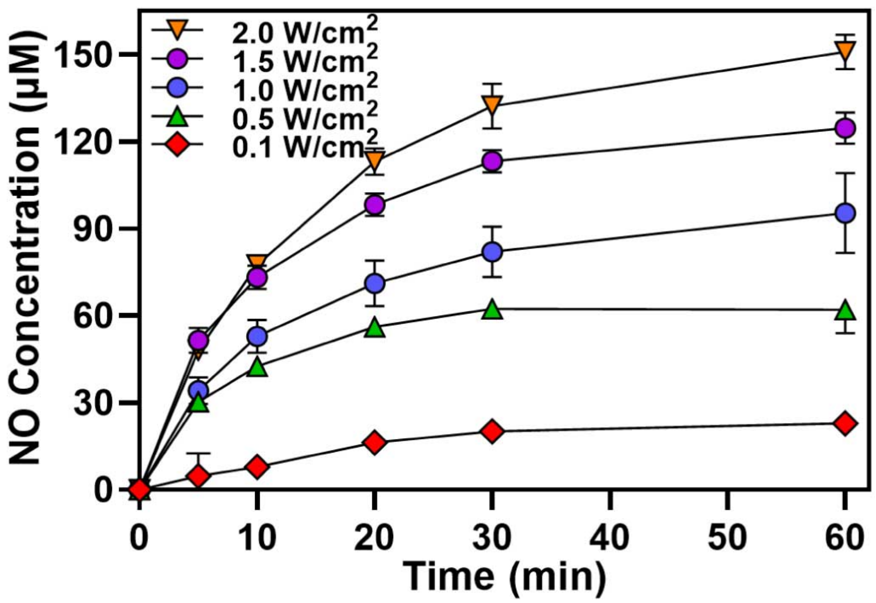

Supplementary Fig. 8 NO generation from T-BD NAs (0.548 mg/mL of DiR and 0.452 mg/mL of BNN6) with different laser power density (808 nm), Data are presented as mean ± SD (n=3 independent experiments) Source data are provided as a Source Data file.

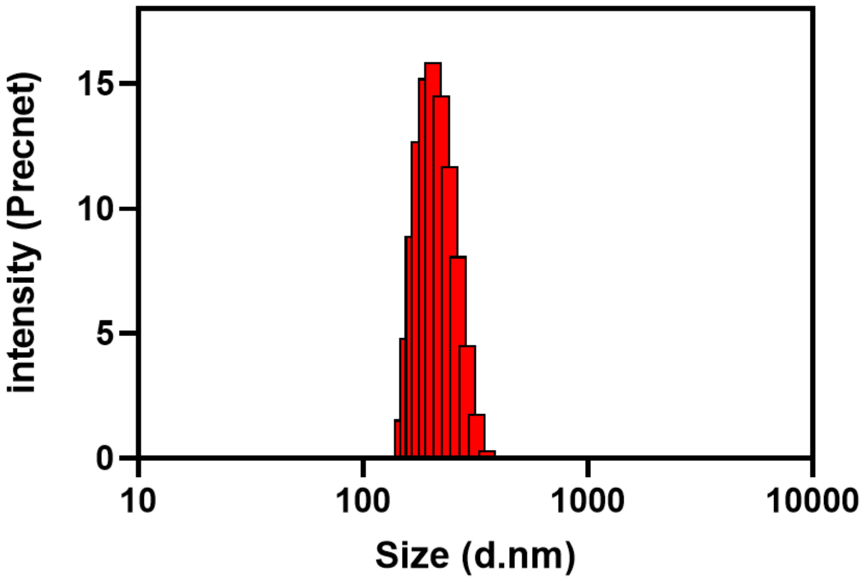

Supplementary Fig. 9 Particle size of PLGA-BD NPs.

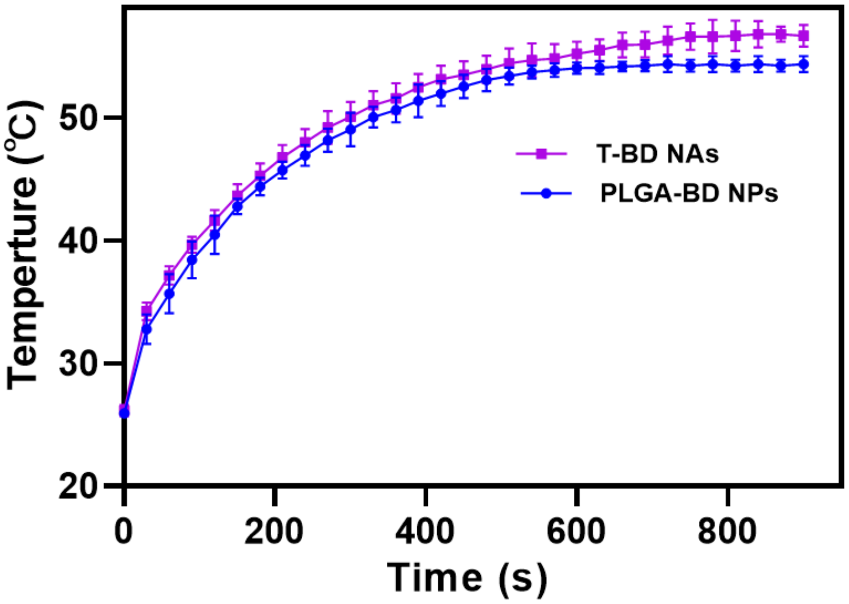

Supplementary Fig. 10 Photothermal curves of T-BD NAs and PLGA-BD NPs under 808 nm laser irradiation (2W/cm^2), Data are presented as mean ± SD (n=3 independent experiments) Source data are provided as a Source Data file.

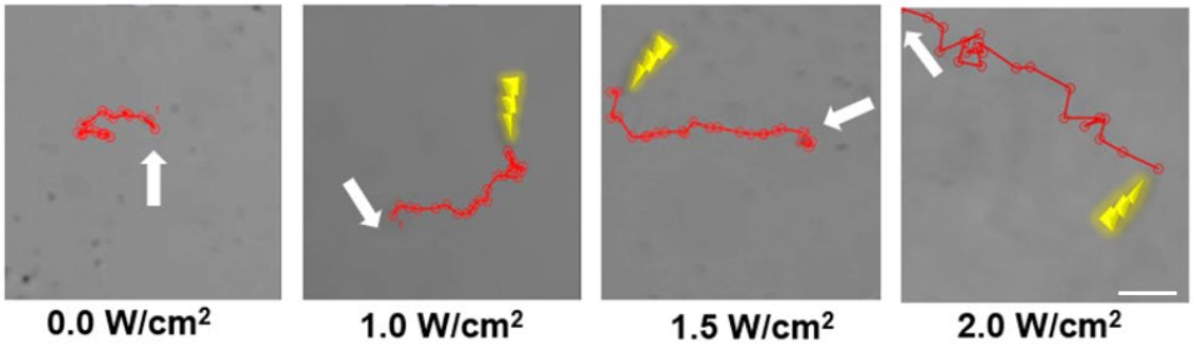

Supplementary Fig. 11 Movement trajectories of T-BD NAs recorded by a fluorescence microscope with Nikon camera under 808 nm laser irradiation with different power intensity from 0 W/cm^2 to 2 W/cm^2, scale bar = 1 μm. Experiment was repeated three times independently with similar results.

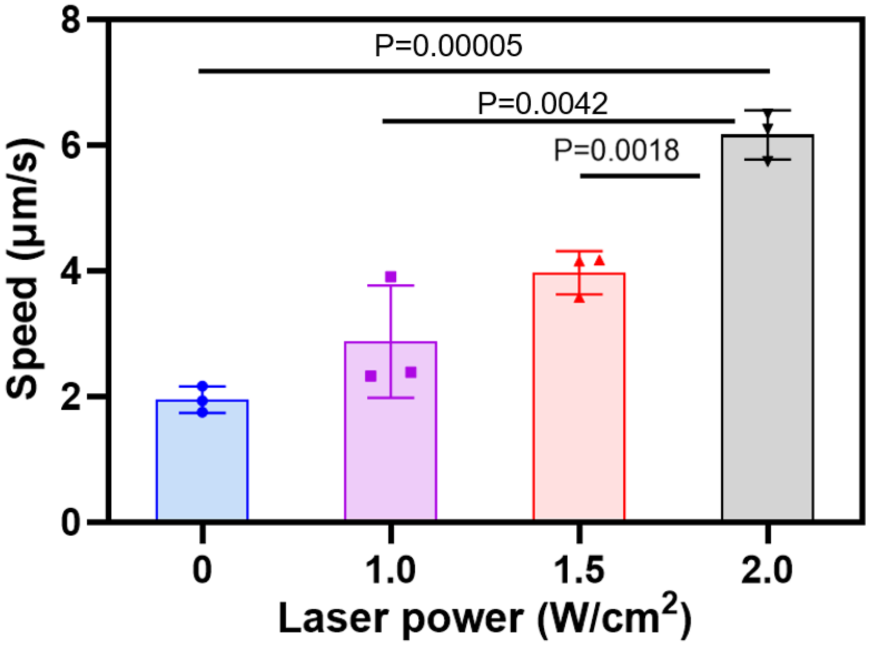

Supplementary Fig. 12 Motion speed of T-BD NAs in PBS (pH 7.4) under 808 nm laser irradiation with different power intensity from 0 W/cm^2 to 2 W/cm^2, Data are presented as mean ± SD (n=3 independent experiments) Source data are provided as a Source Data file.

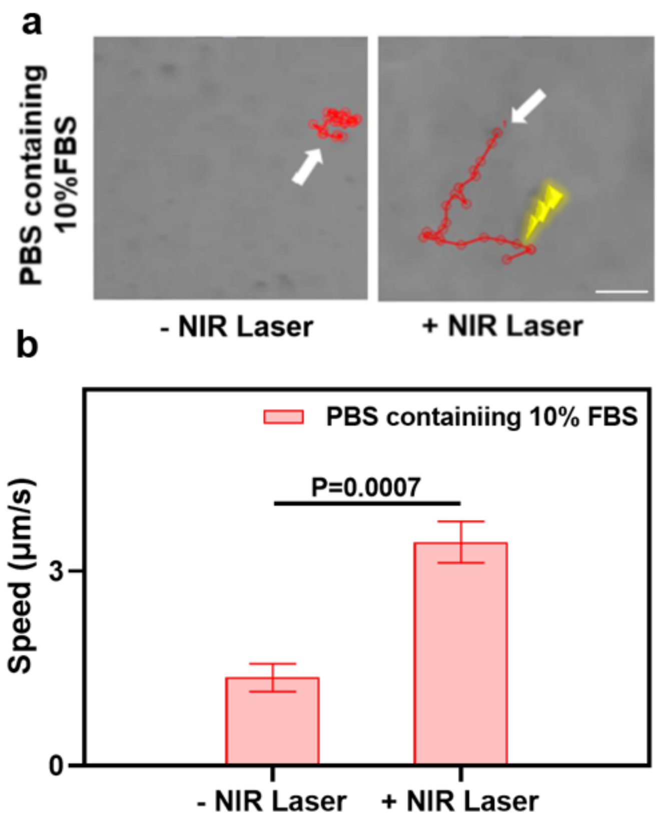

Supplementary Fig. 13 (**a**) Movement trajectories and (**b**) motion speed of T-BD NAs in PBS (pH 7.4) containing 10% FBS under 808 nm laser irradiation (2 W/cm^2, 60 s), scale bar = 1 μm. Data are presented as mean ± SD (n=3 independent experiments) Source data are provided as a Source Data file.

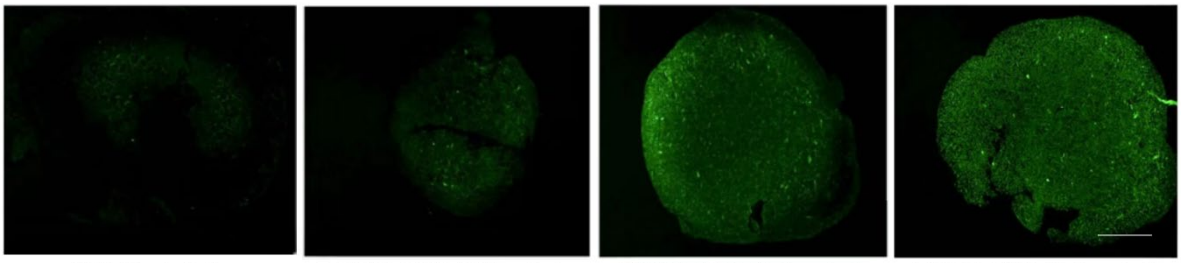

Supplementary Fig. 14 Fluorescence imaging of small red thrombus sections incubated with free C-6, C6/N-BD NAs, C6/B-BD NAs or C6/T-BD NAs under 808 nm laser irradiation (2 W/cm^2, 15 min). scale bar = 1 μm. Experiment was repeated three times independently with similar results.

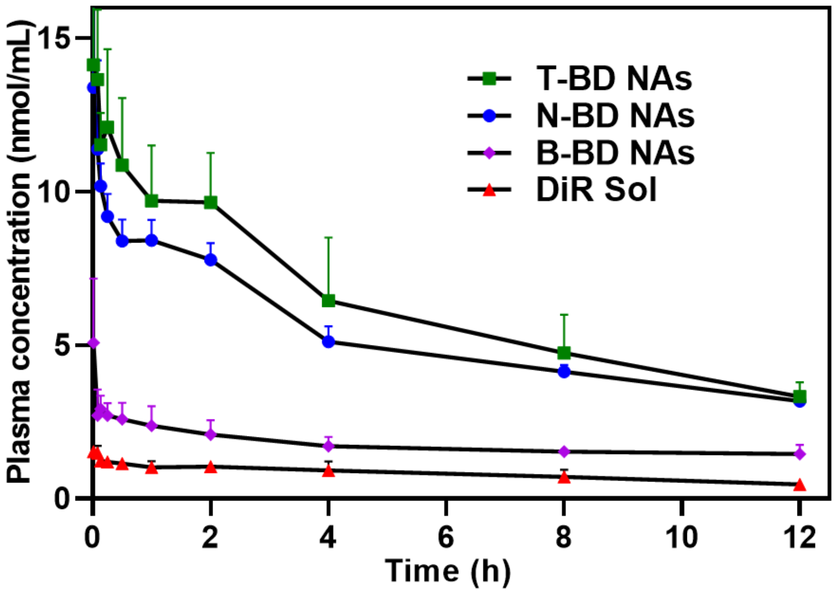

Supplementary Fig. 15 Pharmacokinetic profiles of DiR Sol, B-BD NAs, T-BD NAs, and N-BD NAs after intravenous administration at an equivalent DiR dose of 5 mg/kg, n=5. Data are presented as mean ± SD (n=5 rats) Source data are provided as a Source Data file.

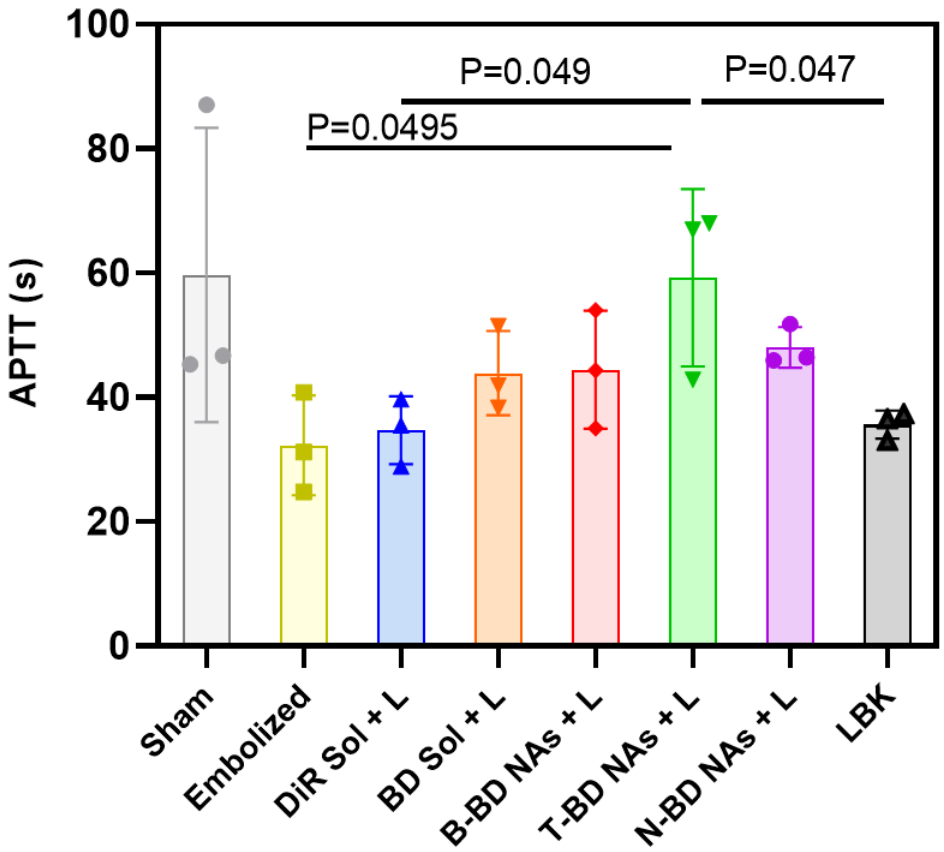

Supplementary Fig. 16 The coagulation indicators of activated partial thromboplastin time (APTT) in $FeCl_3$-induced rat carotid arterial thrombosis model after different treatments (DiR equivalent dose 5 mg/kg, n=3). Data are presented as mean ± SD. Source data are provided as a Source Data file.

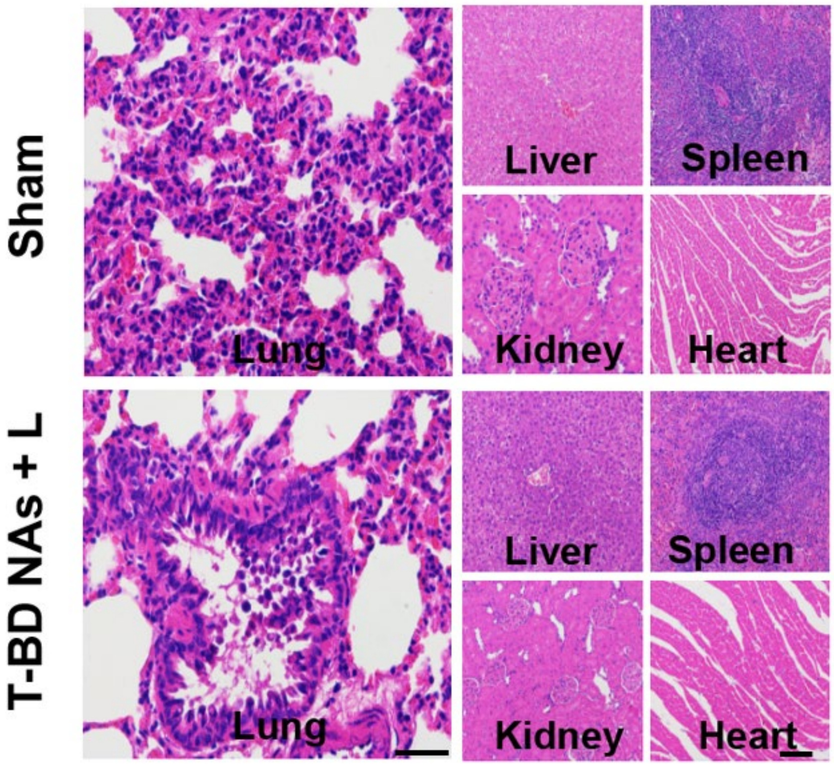

Supplementary Fig. 17 H&E staining sections of the major organs (heart, liver, spleen, lung, and kidney) of rats after treatment with T-BD NAs for 14 days in $FeCl_3$-induced rat carotid arterial thrombosis model under laser irradiation (2.0 W/cm^2, 15 min), scale bar = 100 μm. The Sham rats were used as control. Experiment was repeated three times independently with similar results.

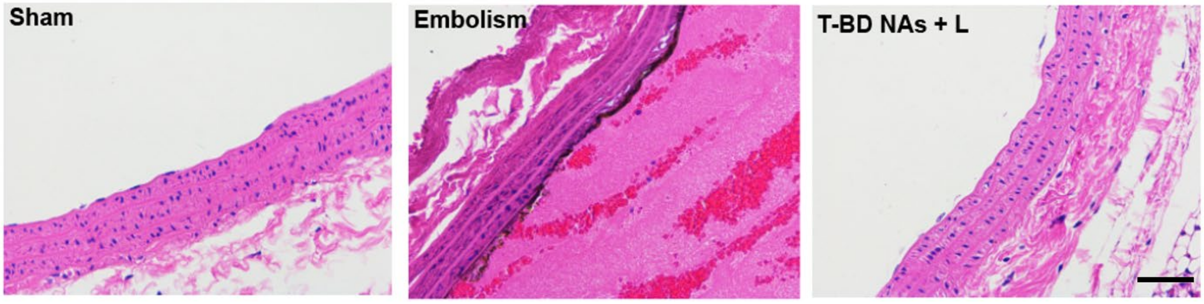

Supplementary Fig. 18 H&E staining sections of carotid artery in rats after various treatment: Sham group, Embolism group, and T-BD NAs + L group (808 nm laser irradiation, 2.0 W/cm^2, 15 min), scale bar = 100 μm. Experiment was repeated three times independently with similar results.

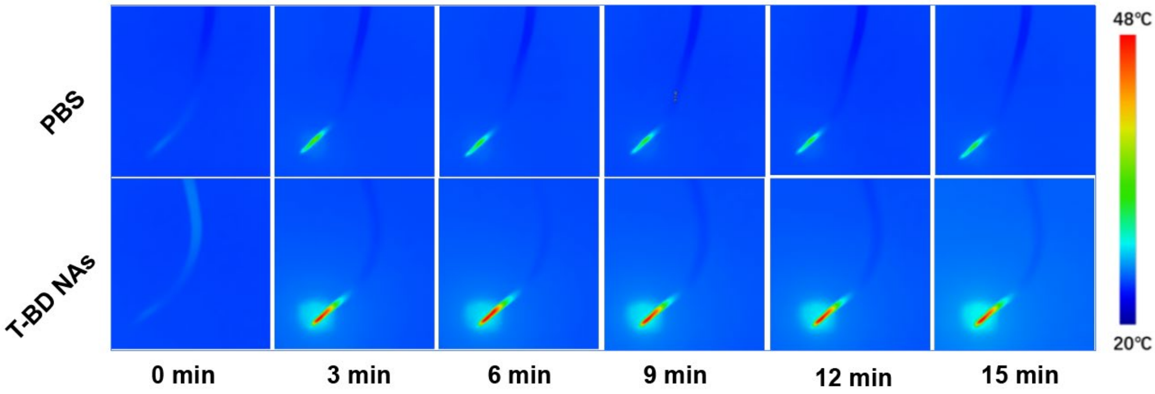

Supplementary Fig. 19 In vivo photothermal imaging of T-BD NAs in the embolized tails.

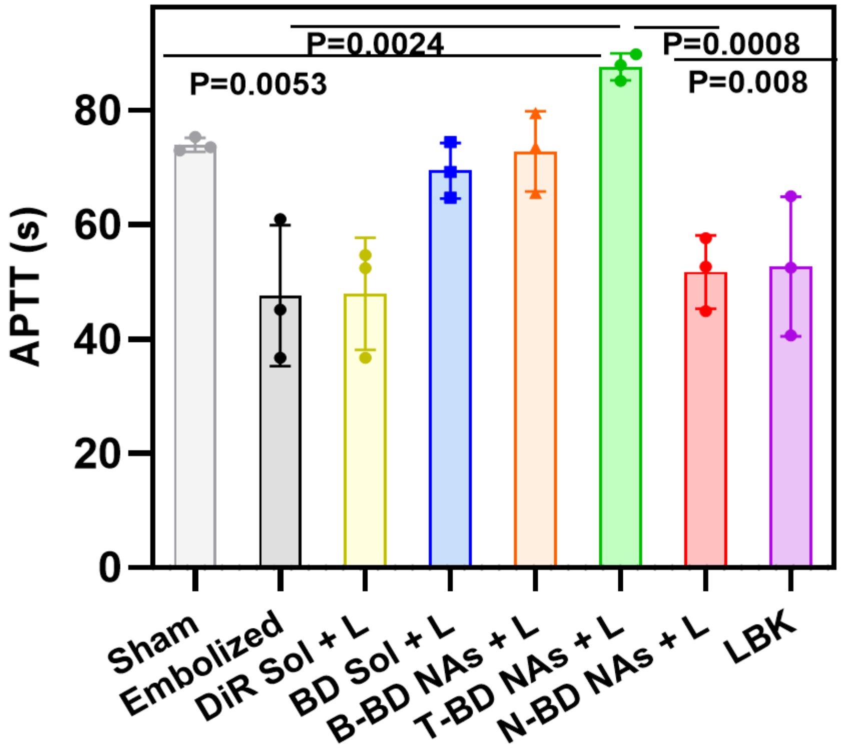

Supplementary Fig. 20 The coagulation indicators of activated partial thromboplastin time (APTT) after different treatments in mouse tail thrombosis model (DiR equivalent dose 5 mg/kg, n=3). Data are presented as mean ± SD (n=5 rats) Source data are provided as a Source Data file.

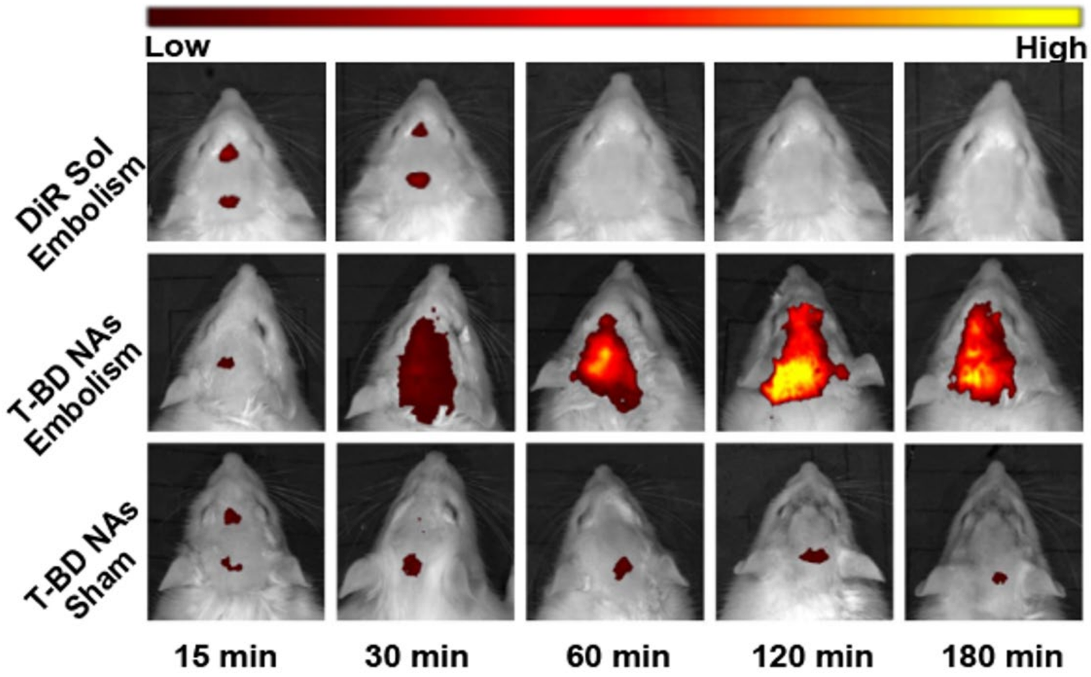

Supplementary Fig. 21 Brain fluorescence imaging of T-BD NAs and DiR Sol in the MCAO model rats and health rats (Sham) after intravenous injection at an equivalent DiR dose of 5 mg/kg.

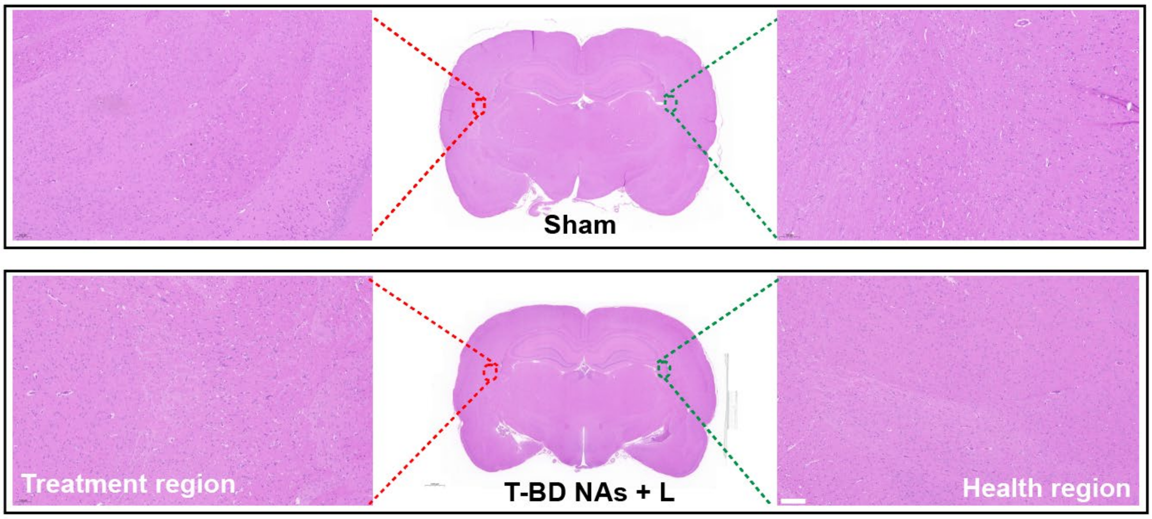

Supplementary Fig. 22 H&E staining sections of the brain tissues in the Sham group and T-BD NAs + L group (2 W/cm^2, 15 min), scale bar = 100 μm. Experiment was repeated three times independently with similar results.

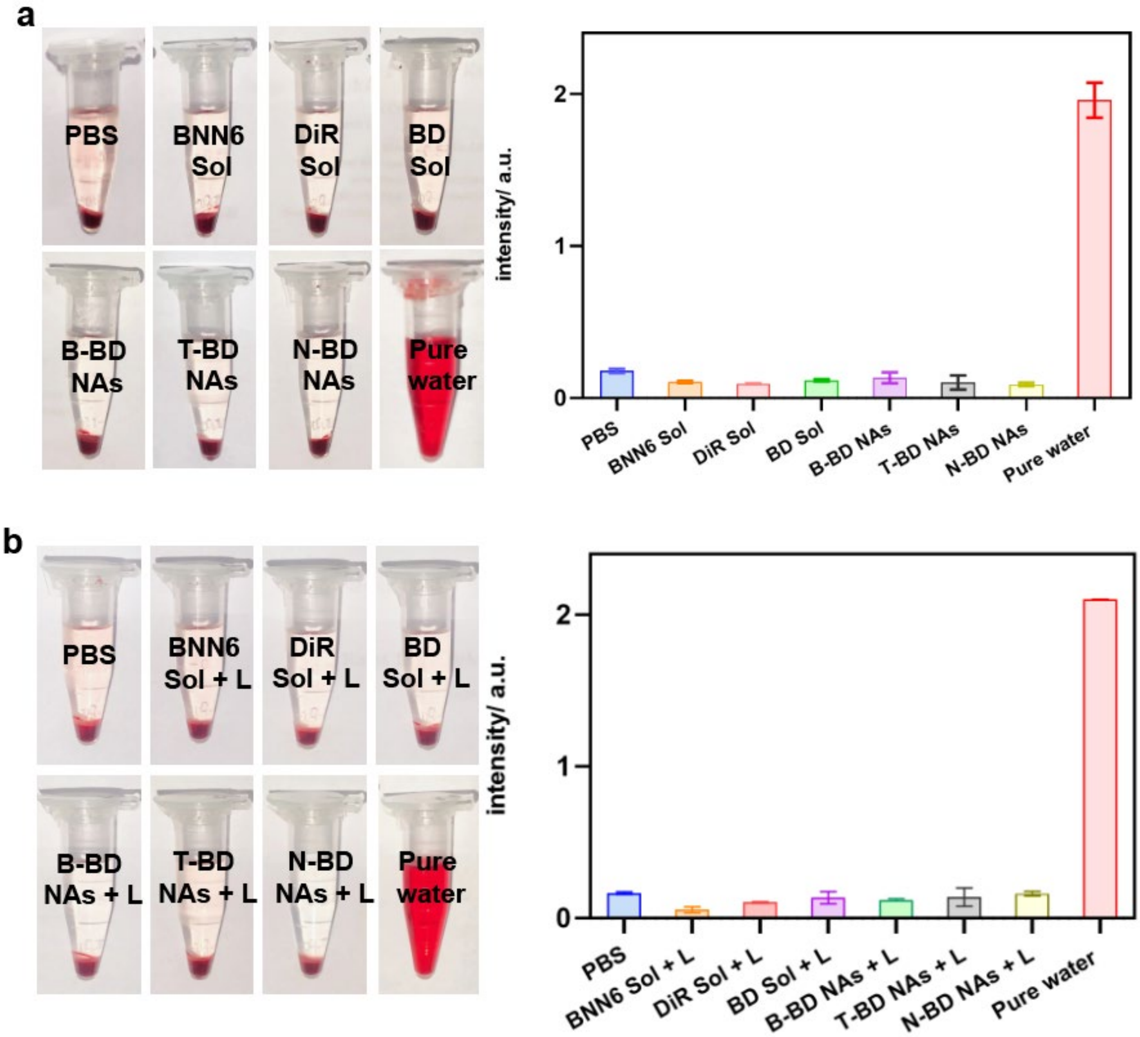

Supplementary Fig. 23 (**a**) Hemolysis results and quantitative analysis of hemoglobin content in the supernatants without laser irradiation. (**b**) Hemolysis images and quantitative analysis of hemoglobin content in the supernatants with the laser irradiation (2 W/cm^2, 5 min). Data are presented as mean ± SD (n=3 independent experiments) Source data are provided as a Source Data file.

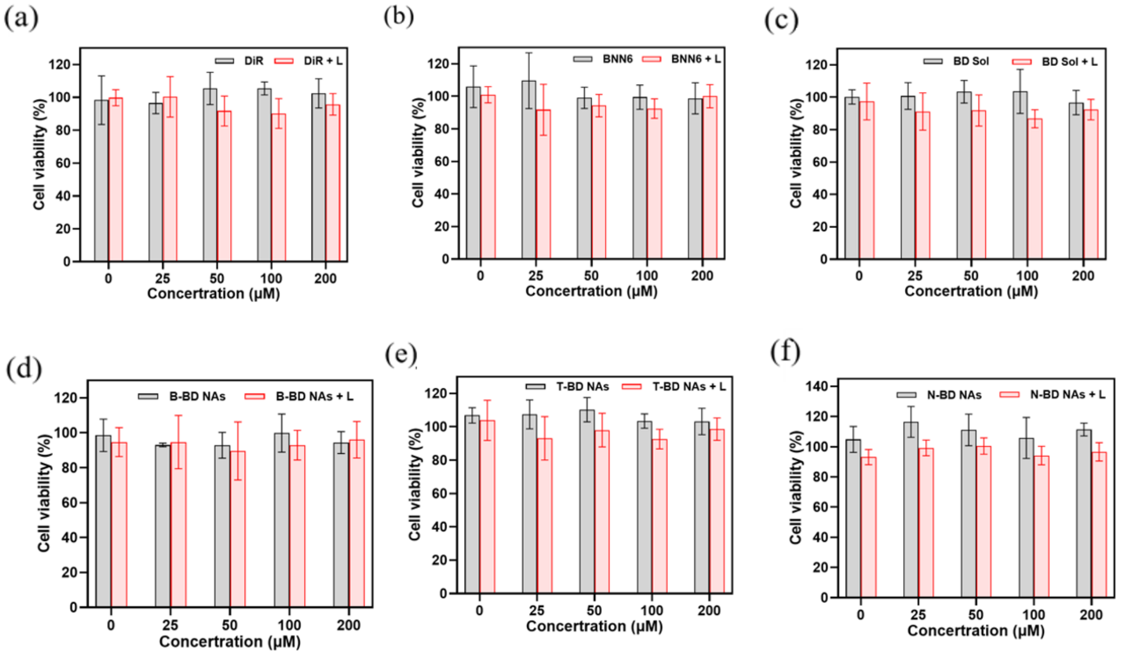

Supplementary Fig. 24 Cell viability of HUVEC after incubation with various concentrations of (**a**) DiR Sol, (**b**) BNN6 Sol, (**c**) BD Sol, (**d**) B-BD NAs, (**e**) T-BD NAs or (**f**) N-BD NAs for 48 h with/without laser irradiation (2 W/cm^2, 5 min). Data are presented as mean ± SD (n=3 biologically independent cells) Source data are provided as a Source Data file.

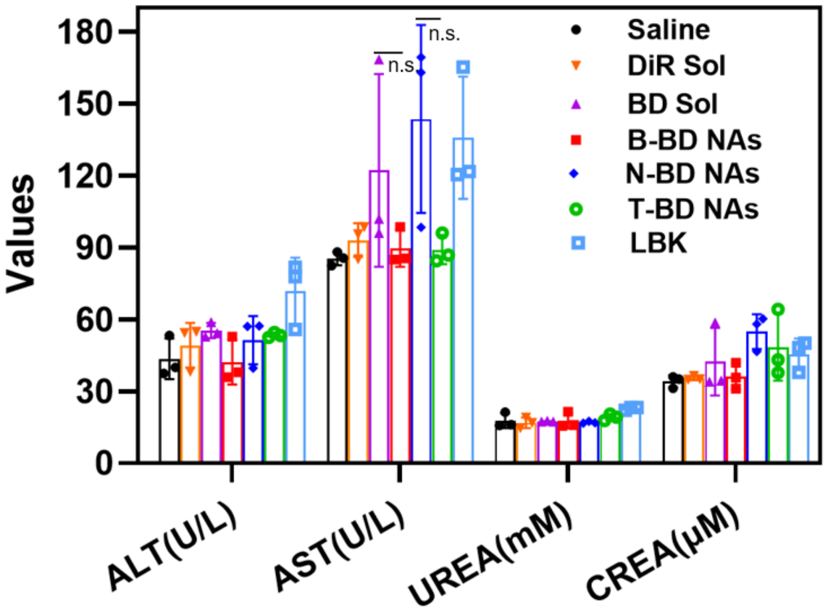

Supplementary Fig. 25 Hepatorenal function parameters of the rats after different treatments (n=3). Data are presented as mean ± SD. Source data are provided as a Source Data file.

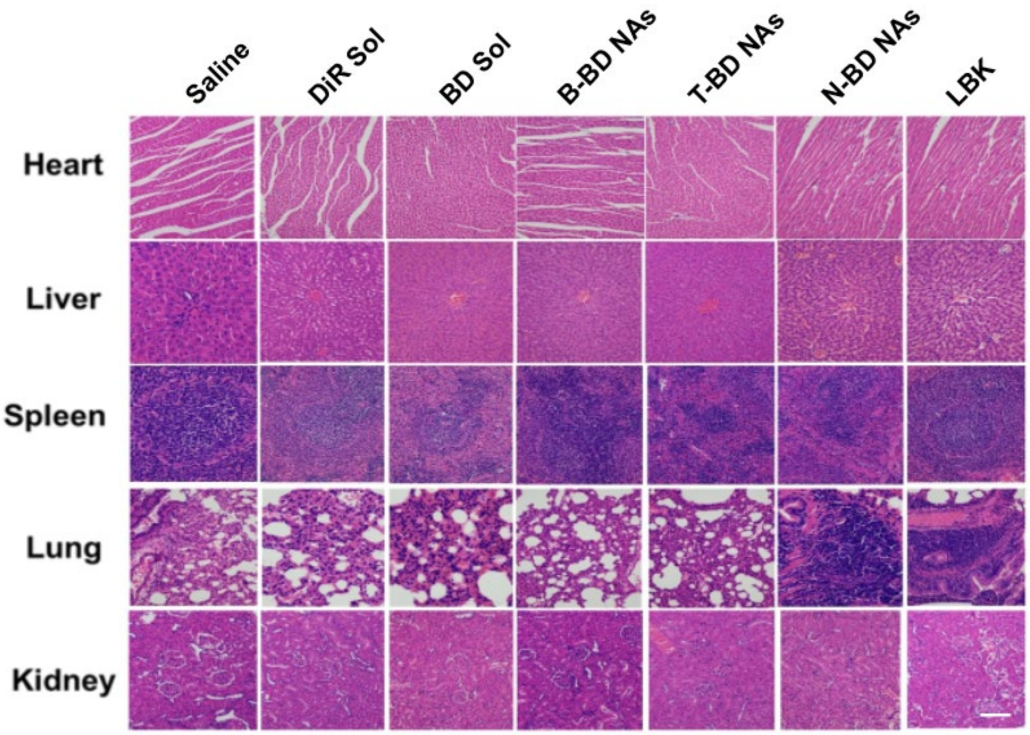

Supplementary Fig. 26 H&E staining sections of the heart, liver, spleen, lung, and kidney in the MCAO rats after different treatments, scale bar = 100 μm. Experiment was repeated three times independently with similar results.

Supplementary Tab. 1 Characterization of B-BD NAs at different molar ratios of DiR and BNN6.

Formulations (DiR/BNN6)	Size[a] (nm)	PDI[b]
10:1	137.7 ± 1.8	0.06 ± 0.04
8:1	138.8 ± 6.3	0.13 ± 0.03
5:1	135.7 ± 7.2	0.11 ± 0.01
3:1	128.3 ± 1.1	0.16 ± 0.05
1:1	91.3 ± 2.5	0.09 ± 0.01
1:3	76.1 ± 0.8	0.12 ± 0.01
1:5	77.9 ± 7.4	0.26 ± 0.01
1:8	176.4 ± 23.1	0.34 ± 0.02
1:10	168.7 ± 16.7	0.47 ± 0.14

[a)] Mean diameters of B-BD NAs measured by DLS. [b)] Polydispersity index of B-BD NAs.

Supplementary Tab. 2 Characterization of B-BD NAs, T-BD NAs and N-BD NAs.

NAs	Size[a] (nm)	PDI[b]	Zeta[a] (mV)	Drug loading rates[c]	
				DiR	BNN6
B-BD NAs	76.1 ± 0.8	0.12 ± 0.01	18.33 ± 0.46	54.8%	45.2%
T-BD NAs	105.0 ± 9.2	0.11 ± 0.02	-7.65 ± 2.17	41.1%,	33.9%
N-BD NAs	118.7 ± 1.3	0.33 ± 0.02	-5.80 ± 1.31	45.4%	29.4%

a) Mean diameters and Zeta potential of NAs measured by DLS. b) Polydispersity index of NAs. c)Drug loading rate of NAs.

Supplementary Tab. 3 Pharmacokinetic parameters of DiR Sol, B-BD NAs, T-BD NAs, and N-BD NAs (n=5).

Formulations	$C_{1\ h}$ [a)]	$AUC_{0\text{-}12\ h}$ [b)]
DiR Sol	1.01 ± 0.21	9.720 ±1.02
B-BD NAs	2.38 ± 0.64	21.06 ± 1.25
T-BD NAs	9.71 ± 1.80	75.23 ± 6.28
N-BD NAs	8.42 ± 0.67	63.01 ±1.57

[a)] The plasma concentration at 1 h (nmol/mL). [b)] Area under the plasma concentration-time curve (nmol/mL*h).
