# Supplementary material for: Molecularly self‐fueled nano-penetrator for nonpharmaceutical treatment of thrombosis and ischemic stroke
Source: Nat Commun. 2023 Jan 17;14:255. doi: 10.1038/s41467-023-35895-5 (PMC9845202; doi:10.1038/s41467-023-35895-5)
Supplement: Supplementary file 2 — Description of Additional Supplementary Files [file 41467_2023_35895_MOESM2_ESM.pdf]

Description of Supplementary Information

File Name:

Supplementary Movie 1

Description: Movement trajectories movie of T-BD NAs recorded by a fluorescence microscope with Nikon camera under 808 nm laser irradiation with different power intensity from 0 W/cm^2 to 2 W/cm^2, (60 s).

Supplementary Movie 2

Description: Movement trajectories movie of T-BD NAs and PLGA-BD NAs incubated in PBS (pH 7.4) at the same dose of DiR/BNN6 under laser irradiations, (0 W/cm2 and 2 W/cm2, 60 s).
