# Supplementary material for: Molecularly self‐fueled nano-penetrator for nonpharmaceutical treatment of thrombosis and ischemic stroke
Source: Nat Commun. 2023 Jan 17;14:255. doi: 10.1038/s41467-023-35895-5 (PMC9845202; doi:10.1038/s41467-023-35895-5)
Supplement: Supplementary file 5 — Reporting Summary [file 41467_2023_35895_MOESM5_ESM.pdf]

nature portfolio

Corresponding author(s): Cong Luo

Last updated by author(s): Jan 1, 2023

Reporting Summary

Nature Portfolio wishes to improve the reproducibility of the work that we publish. This form provides structure for consistency and transparency in reporting. For further information on Nature Portfolio policies, see our Editorial Policies and the Editorial Policy Checklist.

Statistics

For all statistical analyses, confirm that the following items are present in the figure legend, table legend, main text, or Methods section.

n/a	Confirmed	
☐	☒	The exact sample size (*n*) for each experimental group/condition, given as a discrete number and unit of measurement
☐	☒	A statement on whether measurements were taken from distinct samples or whether the same sample was measured repeatedly
☐	☒	The statistical test(s) used AND whether they are one- or two-sided<br>*Only common tests should be described solely by name; describe more complex techniques in the Methods section.*
☐	☒	A description of all covariates tested
☐	☒	A description of any assumptions or corrections, such as tests of normality and adjustment for multiple comparisons
☐	☒	A full description of the statistical parameters including central tendency (e.g. means) or other basic estimates (e.g. regression coefficient) AND variation (e.g. standard deviation) or associated estimates of uncertainty (e.g. confidence intervals)
☐	☒	For null hypothesis testing, the test statistic (e.g. *F*, *t*, *r*) with confidence intervals, effect sizes, degrees of freedom and *P* value noted<br>*Give P values as exact values whenever suitable.*
☐	☒	For Bayesian analysis, information on the choice of priors and Markov chain Monte Carlo settings
☐	☒	For hierarchical and complex designs, identification of the appropriate level for tests and full reporting of outcomes
☒	☐	Estimates of effect sizes (e.g. Cohen's *d*, Pearson's *r*), indicating how they were calculated

Our web collection on statistics for biologists contains articles on many of the points above.

Software and code

Policy information about availability of computer code

Data collection	Bruker AV-400; FT-MS(Bruker): ftmsControl 2.1; Zetasizer (Nano ZS, Malvern Co., UK);TEM, HITACHI, HT7700, Japan; IVIS Lumina Series III;Fotric 226;Confocal laser scanning microscopy: NIS 4.13,;multimode microreader (Thermo Scientific, USA)
Data analysis	MestReC 4.9.9.9; ftmsControl 2.1 ;Bruker Compass DataAnalysis 4.4; GraphPad prism 8.0; DAS 2.1.1; ImageJ 1.8.0

For manuscripts utilizing custom algorithms or software that are central to the research but not yet described in published literature, software must be made available to editors and reviewers. We strongly encourage code deposition in a community repository (e.g. GitHub). See the Nature Portfolio guidelines for submitting code & software for further information.

Data

Policy information about availability of data

All manuscripts must include a data availability statement. This statement should provide the following information, where applicable:

- Accession codes, unique identifiers, or web links for publicly available datasets
- A description of any restrictions on data availability
- For clinical datasets or third party data, please ensure that the statement adheres to our policy

All data supporting the findings of this study are available within the Article, Supplementary Information or Source Data file. The source data underlying Figure 2c-d,

g-h, j-l, Figure 3c-d, f-g,i-k, Figure 4c, f, h , Figure 5c-d, f, i, Figure 6c-d, h, Figure 7c-d,f, h, supplementary Fig 4, 5b, 6, 7, 8, 9, 10, 12, 13b, 15, 16, 20, 23, 24, and 25 have been deposited in the Figshare database (DOI: 10.6084/m9.figshare.21801139.v1)

Human research participants

Policy information about studies involving human research participants and Sex and Gender in Research.

Reporting on sex and gender	not applicable
Population characteristics	not applicable
Recruitment	not applicable
Ethics oversight	not applicable

Note that full information on the approval of the study protocol must also be provided in the manuscript.

Field-specific reporting

Please select the one below that is the best fit for your research. If you are not sure, read the appropriate sections before making your selection.

☒ Life sciences ☐ Behavioural & social sciences ☐ Ecological, evolutionary & environmental sciences

For a reference copy of the document with all sections, see nature.com/documents/nr-reporting-summary-flat.pdf

Life sciences study design

All studies must disclose on these points even when the disclosure is negative.

Sample size	Sample size was selected from the general sample size in the reference. Increasing the number of parallelisms could reduce the accidental error and improve the precision of the experiment. According to the statistical principle, when 'n' is increased, the corresponding precision will also be improved, and then three to four times is the most appropriate. For in vitro experiments, n=3 was selected in order to measure the distribution closer to the true distribution on the premise of cost saving. For in vivo experiments, n=5 was selected to obtain five biological repetitions in consideration of animal individual differences and the 4R principle for the credibility of the results (Nature Communications, 2022, 13(1): 1-15).
Data exclusions	No data were excluded from the analyses.
Replication	We confirmed that all repeated attempts were successful. Experiment repeat numbers are reported in Figure Legends.
Randomization	Samples were randomly allocated into experimental groups.
Blinding	The investigators were blinded to group allocation during data collection and analysis.

Reporting for specific materials, systems and methods

We require information from authors about some types of materials, experimental systems and methods used in many studies. Here, indicate whether each material, system or method listed is relevant to your study. If you are not sure if a list item applies to your research, read the appropriate section before selecting a response.

Materials & experimental systems

n/a	Involved in the study	
☒	☐	Antibodies
☐	☒	Eukaryotic cell lines
☒	☐	Palaeontology and archaeology
☐	☒	Animals and other organisms
☒	☐	Clinical data
☒	☐	Dual use research of concern

Methods

n/a	Involved in the study	
☒	☐	ChIP-seq
☒	☐	Flow cytometry
☒	☐	MRI-based neuroimaging

Eukaryotic cell lines

Policy information about cell lines and Sex and Gender in Research

Cell line source(s)	HUVEC were obtained from Shanghai Qingqi Biotechnology Development Co., Ltd
Authentication	HUVEC lines validation using short tandem repeat (STR) markers were performed by Shanghai Qingqi Biotechnology Development Co., Ltd. Sample DNA was extracted using Axygen's genome extraction kit and amplified using a 10-STR amplification protocol (D4S2408 as the human locus). STR loci were detected on an ABI model 3730XL genetic analyzer. Data were analyzed using Gene Mapper ID 3.2 software (Applied Biosystems). Appropriate positive and negative controls were run and confirmed for each sample submitted.
Mycoplasma contamination	All cell lines tested negative for mycoplasma contamination.
Commonly misidentified lines (See ICLAC register)	No commonly misidentified cells lines were used in the study.

Animals and other research organisms

Policy information about studies involving animals; ARRIVE guidelines recommended for reporting animal research, and Sex and Gender in Research

Laboratory animals	Sprague-Dawley rats (male, 6-week old) and KM mice (male, 11-week old) were supplied by the Animal Center of Shenyang Pharmaceutical University (Shenyang, Liaoning, China). The living environment of animals were maintained at a temperature of ~25 ° C with a 12 h light/dark cycle, with free access to standard food and water.
Wild animals	The study did not involve wild animals.
Reporting on sex	Although we have used single-sex animals in our research, we think that the research results were not only applicable to single sex. For instance, male rats were used in the pharmacokinetic experiment, and gender had no influence on the pharmacokinetic results.
Field-collected samples	The study did not involve samples collected from the field.
Ethics oversight	All the animal experiments were conducted according to the Guidelines for the Care and Use of Laboratory Animals approved by the Institutional Animal Ethical Care Committee (IAEC) of Shenyang Pharmaceutical University.

Note that full information on the approval of the study protocol must also be provided in the manuscript.
